# GABAergic/Glycinergic and Glutamatergic Neurons Mediate Distinct Neurodevelopmental Phenotypes of *STXBP1* Encephalopathy

**DOI:** 10.1523/JNEUROSCI.1806-23.2024

**Published:** 2024-02-15

**Authors:** Joo Hyun Kim, Wu Chen, Eugene S. Chao, Armando Rivera, Heet Naresh Kaku, Kevin Jiang, Dongwon Lee, Hongmei Chen, Jaimie M. Vega, Teresa V. Chin, Kevin Jin, Kelly T. Nguyen, Sheldon S. Zou, Zain Moin, Shawn Nguyen, Mingshan Xue (薛名杉)

**Affiliations:** ^1^Department of Neuroscience, Baylor College of Medicine, Houston, Texas 77030; ^2^The Cain Foundation Laboratories, Jan and Dan Duncan Neurological Research Institute at Texas Children’s Hospital, Houston, Texas 77030; ^3^Department of Molecular and Human Genetics, Baylor College of Medicine, Houston, Texas 77030

**Keywords:** synapse, excitation, inhibition, epilepsy, intellectual disability, neurobehavior

## Abstract

An increasing number of pathogenic variants in presynaptic proteins involved in the synaptic vesicle cycle are being discovered in neurodevelopmental disorders. The clinical features of these synaptic vesicle cycle disorders are diverse, but the most prevalent phenotypes include intellectual disability, epilepsy, movement disorders, cerebral visual impairment, and psychiatric symptoms ( Verhage and Sørensen, 2020; Bonnycastle et al., 2021; John et al., 2021; Melland et al., 2021). Among this growing list of synaptic vesicle cycle disorders, the most frequent is *STXBP1* encephalopathy caused by *de novo* heterozygous pathogenic variants in syntaxin-binding protein 1 (STXBP1, also known as MUNC18-1; Verhage and Sørensen, 2020; John et al., 2021). STXBP1 is an essential protein for presynaptic neurotransmitter release. Its haploinsufficiency is the main disease mechanism and impairs both excitatory and inhibitory neurotransmitter release. However, the disease pathogenesis and cellular origins of the broad spectrum of neurological phenotypes are poorly understood. Here we generate cell type-specific *Stxbp1* haploinsufficient male and female mice and show that *Stxbp1* haploinsufficiency in GABAergic/glycinergic neurons causes developmental delay, epilepsy, and motor, cognitive, and psychiatric deficits, recapitulating majority of the phenotypes observed in the constitutive *Stxbp1* haploinsufficient mice and *STXBP1* encephalopathy. In contrast, *Stxbp1* haploinsufficiency in glutamatergic neurons results in a small subset of cognitive and seizure phenotypes distinct from those caused by *Stxbp1* haploinsufficiency in GABAergic/glycinergic neurons. Thus, the contrasting roles of excitatory and inhibitory signaling reveal GABAergic/glycinergic dysfunction as a key disease mechanism of *STXBP1* encephalopathy and suggest the possibility to selectively modulate disease phenotypes by targeting specific neurotransmitter systems.

## Significance Statement

Heterozygous pathogenic variants in *STXBP1* are in the top 5 causes of pediatric epilepsies and one of the most frequent causes of neurodevelopmental disorders. They affect presynaptic neurotransmitter release and a broad spectrum of neurological features common among neurodevelopmental disorders, but the disease pathogenesis and cell types subserving these phenotypes remain unclear. Here we report the distinct roles of GABAergic/glycinergic and glutamatergic neurons in the pathogenesis of *STXBP1* encephalopathy. These results will aid the development of therapeutic interventions by suggesting the potential outcomes of therapeutic strategies that target different neuronal types for treating *STXBP1* encephalopathy.

## Introduction

*STXBP1* encephalopathy, caused by heterozygous pathogenic variants in syntaxin-binding protein 1 (STXBP1, also known as MUNC18-1), is among the most frequent developmental and epileptic encephalopathies (Symonds and McTague, 2020; Verhage and Sørensen, 2020) and neurodevelopmental disorders (Deciphering Developmental Disorders Study, 2015; Kaplanis et al., 2020). All *STXBP1* encephalopathy patients have intellectual disability, and 80–90% of the patients have epilepsy and motor dysfunctions (Stamberger et al., 2016; Abramov et al., 2021; Xian et al., 2022). Other clinical features include developmental delay, autistic traits, hyperactivity, anxiety, and aggressive behaviors (Stamberger et al., 2016; Suri et al., 2017; Xian et al., 2022).

Haploinsufficiency is the major disease mechanism of *STXBP1* encephalopathy because >50% of the mutations are truncating variants (Stamberger et al., 2016; Abramov et al., 2021; Xian et al., 2022), but a dominant-negative mechanism was proposed for a subset of missense variants (Chai et al., 2016; Guiberson et al., 2018). *STXBP1* encephalopathy was successfully modeled in fish and mice. Removing *stxbp1b*, one of the two *STXBP1* homologs in zebrafish, causes spontaneous electrographic seizures (Grone et al., 2016). The first three *Stxbp1* heterozygous knock-out mouse models recapitulate a subset of neurological phenotypes seen in patients (Hager et al., 2014; Miyamoto et al., 2017; Kovačević et al., 2018; Orock et al., 2018). We generated two new mouse *Stxbp1* null alleles, and the heterozygous mice (*Stxbp1^tm1a/+^* and *Stxbp1^tm1d/+^*) show ∼50% reduction in Stxbp1 protein levels in most brain regions (Chen et al., 2020). Both models recapitulate nearly all features of *STXBP1* encephalopathy, as they show early lethality, developmental delay, cognitive impairments, motor dysfunction, anxiety-like behaviors, hyperactivity, aggression, and epileptic seizures including spike-wave discharges (SWDs) and myoclonic seizures (Chen et al., 2020).

Mechanistically, it is well established that STXBP1 is required for synaptic vesicle exocytosis in all neurons (Harrison et al., 1994; Verhage et al., 2000; Weimer et al., 2003). The heterozygous null mutations impair both glutamatergic excitatory and GABAergic inhibitory neurotransmitter release, but the exact synaptic phenotypes differ at different synapses or ages (Toonen et al., 2006; Patzke et al., 2015; Orock et al., 2018; Miyamoto et al., 2019; Chen et al., 2020; Dos Santos et al., 2023). However, the significance of these diverse synaptic phenotypes to neurological impairments remains unclear. To address this crucial question and identify the cellular origins of disease, one approach is to create conditional *Stxbp1* haploinsufficiency in specific neuronal types to determine the impact on neurological functions, as this manipulation selectively affects the presynaptic outputs of targeted neurons. Previous studies showed that *Stxbp1* heterozygous knock-out in dorsal telencephalic excitatory neurons resulted in frequent SWDs and reduced associative memory (Miyamoto et al., 2017, 2019), whereas *Stxbp1* heterozygous knock-out in inhibitory neurons led to involuntary twitches and jumps (Miyamoto et al., 2019) and reduced survival (Kovačević et al., 2018). Thus, these cell type-specific *Stxbp1* heterozygous deletions did not recapitulate the full extent of neurological impairments in *STXBP1* encephalopathy and constitutive *Stxbp1* haploinsufficient mice, which seems to suggest that neither glutamatergic nor GABAergic/glycinergic neurons are critical for the disease pathogenesis. However, these studies did not investigate many neurodevelopmental phenotypes present in the constitutive *Stxbp1* haploinsufficient mice or ascertain the efficacy and specificity of *Stxbp1* conditional deletions. Thus, the significance of synaptic excitatory and inhibitory dysfunctions to the disease pathogenesis remains unclear.

To fill this knowledge gap and define the overall roles of excitation and inhibition in *STXBP1* encephalopathy pathogenesis, we sought to broadly target glutamatergic neurons by *vesicular glutamate transporter 2* (*Vglut2)-ires-Cre* (Vong et al., 2011) and GABAergic/glycinergic neurons by *vesicular inhibitory amino acid transporter (Viaat)-ires-Cre* (Vong et al., 2011) since glycine is often co-released with GABA from some inhibitory neurons (Vaaga et al., 2014). We generated and validated mouse models of *Stxbp1* haploinsufficiency specific to glutamatergic or GABAergic/glycinergic neurons and systematically determined their phenotypes in the three core disease domains—cognitive impairment, epilepsy, and motor dysfunction—as well as psychiatric functions and general health.

## Materials and Methods

### Mice

*Stxbp1* flox mice were generated from a previously described *Stxbp1* knock-out first allele (*tm1a*) that contains a trapping cassette flanked by two *FRT* sites and the exon 7 flanked by two *loxP* sites (Chen et al., 2020). *Stxbp1^tm1a/+^* mice were crossed to *Rosa26-Flpo* mice (Raymond and Soriano, 2007) from the Jackson Laboratory (JAX #012930) to remove the trapping cassette in the germline, resulting in the *Stxbp1* flox allele (*tm1c*). *Stxbp1* flox mice were genotyped by PCR using a pair of primers 5′-TTCCACAGCCCTTTACAGAAAGG-3′ and 5′-ATGTGTATGCCTGGACTCACAGGG-3′ for both wild-type (WT; 352 bp) and *tm1c* (500 bp) alleles. *Stxbp1* flox mice were maintained on the C57BL/6J background by crossing to WT C57BL/6J mice (JAX #000664).

Male heterozygous C57BL/6J-congenic *Viaat-ires-Cre* (JAX #028862; Vong et al., 2011) mice were crossed with female heterozygous *Stxbp1* flox mice (*Stxbp1^f/+^*) to generate WT, *Stxbp1^f/+^*, *Viaat^Cre/+^*, and *Stxbp1^f/+^;Viaat^Cre/+^* mice. Male heterozygous C57BL/6J-congenic *Vglut2-ires-Cre* mice (JAX #028863; Vong et al., 2011) were crossed with female *Stxbp1^f/+^* mice to generate WT, *Stxbp1^f/+^*, *Vglut2^Cre/+^*, and *Stxbp1^f/+^;Vglut2^Cre/+^* mice. Male hemizygous *Dlx5/6-Cre* mice (JAX #028863; Monory et al., 2006) on a C57BL/6J background were crossed with female heterozygous C57BL/6J-congenic *Rosa26-CAG-LSL-tdTomato* mice (Ai14 line, JAX #007914 or Ai9 line, JAX #007909; Madisen et al., 2010) to generate *Dlx5/6-Cre^Tg/+^;Rosa26^tdTomato/+^* mice or with female *Stxbp1^f/+^* mice to generate *Stxbp1^f/+^;Dlx5/6-Cre^Tg/+^* and *Dlx5/6-Cre^Tg/+^* mice. Male homozygous *Pv-ires-Cre* mice (JAX #017320; Hippenmeyer et al., 2005) on the C57BL/6J background, male homozygous *Sst-ires-Cre* mice (JAX #013044; Taniguchi et al., 2011) on a C57BL/6;129S4 background, or male hemizygous *Htr3a-Cre* mice (NO152 line, MMRRC #036680-UCD; Gerfen et al., 2013; Miyoshi et al., 2015) on a FVB;C57BL6;129;Swiss;CD1 background were crossed with female *Stxbp1^f/+^* mice to generate *Stxbp1^f/+^;Pv^Cre/+^* and *Pv^Cre/+^* mice, *Stxbp1^f/+^;Sst^Cre/+^* and *Sst^Cre/+^* mice, or *Stxbp1^f/+^;Htr3a-Cre^Tg/+^* and *Htr3a-Cre^Tg/+^* mice, respectively. Male white BALB/cAnNTac mice (Taconic #BALB-M) or BALB/cJ (JAX #000651) were used for the resident–intruder test.

Mice were housed in an Association for Assessment and Accreditation of Laboratory Animal Care International-certified animal facility on a 14/10 h light/dark cycle. All procedures to maintain and use mice were performed in strict accordance with the recommendations in the Guide for the Care and Use of Laboratory Animals of the National Institutes of Health and were approved by the Institutional Animal Care and Use Committee at Baylor College of Medicine (protocol AN-6544).

### Western blots

Western blot analyses were performed according to the protocols published previously (Chen et al., 2020) with modifications. Proteins were extracted from the entire brains of newborn pups or different brain regions of adult mice in the lysis buffer that contained 50 mM Tris-HCl, pH 7.6, 150 mM NaCl, 1 mM EDTA, 1% Triton X-100, 0.5% Na-deoxycholate, 0.1% SDS, and 1 tablet of cOmplete, Mini, EDTA-free Protease Inhibitor Cocktail (Roche, catalog #SKU 11836170001) in 10 ml buffer. Prior to sodium dodecyl-sulfate polyacrylamide gel electrophoresis (SDS-PAGE), protein samples were incubated in the Laemmli buffer at 98°C for 10 min for measuring Stxbp1, at 55°C for 10 min for measuring Vglut2, or sonicated on ice without heating up for measuring Viaat. Stxbp1 was detected by a rabbit antibody against the N-terminal residues 58–70 (Abcam, catalog #ab3451, lot #GR79394-18 at 1:2,000 or 1:5,000 dilution, lot #GR3261450-6 at 1:50,000 dilution, or lot #GR3414210-5 at 1:60,000 dilution) or a rabbit antibody against the C-terminal residues 580–594 (Synaptic Systems, catalog #116002, lot #116002/15, 1:2,000 or 1:5,000 dilution). Viaat was detected by a mouse antibody (Synaptic Systems, catalog #131011, lot #1-102, 1:5,000 dilution) and Vglut2 by a rabbit antibody (Synaptic Systems, catalog #135403, lot #4-82, 1:5,000 dilution). Gapdh was detected by a rabbit antibody (Santa Cruz Biotechnology, catalog #sc-25778, lot #A0515 at 1:300 or 1:1,000 dilution; GeneTex, catalog #GTX100118, lot #43929 at 1:10,000 dilution; or GeneTex, catalog #GTX637966, lot #44977 at 1:10,000 dilution) or a mouse antibody (MilliporeSigma, catalog #CB1001-500UG, lot #3725987 at 1:10,000 dilution). Primary antibodies were detected by a goat anti-rabbit antibody conjugated with IRDye 680LT (LI-COR Biosciences, catalog #925-68021, lot #C40917-01, D20119-11, or D30425-21 at 1:20,000 dilution) or a goat anti-mouse antibody conjugated with IRDye 800CW (LI-COR Biosciences, catalog #925-32210, lot #D30418-21 at 1:20,000 dilution). Proteins were visualized and quantified using an Odyssey CLx Imager and Image Studio Lite version 5.2.5 (LI-COR Biosciences). Stxbp1, Viaat, and Vglut2 levels were normalized by the Gapdh levels. The results from the two Stxbp1 antibodies were averaged for mice tested by both antibodies.

### Double fluorescence in situ hybridization and imaging

Digoxigenin (DIG)-labeled RNA antisense probes against mouse *Stxbp1* or *tdTomato* and fluorescein (FITC)-labeled RNA antisense probes against mouse *Vglut1* (*Slc17a7*), *Vglut2* (*Slc17a6*), or *Gad1* were generated by in vitro transcription using cDNA templates and RNA DIG- or FITC-labeling kits (Sigma, catalog #11277073910 or 11685619910, respectively). The DNA templates were generated by PCR amplification from a plasmid pCMV-SPORT6-Stxbp1a (GenBank: BC031728.1, Transomic Technologies) for the *Stxbp1* probe, or from mouse brain cDNA for the *Vglut1*, *Vglut2*, *Gad1*, and *tdTomato* probes, with a SP6 promoter (ATTTAGGTGACACTATAG) or a T3 promoter (AATTAACCCTCACTAAAGGG) added at the 5′ end of the PCR forward primers and a T7 promoter (TAATACGACTCACTATAGGG) at the 5′ end of the PCR reverse primers. The sequences of *Stxbp1*, *Vglut1*, *Vglut2*, and *tdTomato* probes were from Allen Brain Atlas (http://mouse.brain-map.org) and *Gad1* from Eurexpress (http://www.eurexpress.org/ee/). The probe sequences are provided in Extended Data List 1-1.

10.1523/JNEUROSCI.1806-23.2024.d1-1 Extended Data List 1-1 **Double fluorescence *in situ* hybridization probe sequences** The sequences of the *Stxbp1*, *Gad1*, *Vglut1*, *Vglut2*, and *tdTomato* probes are provided. Download Extended Data List 1-1, DOCX file.

Double fluorescence in situ hybridization (DFISH) was performed by the RNA In Situ Hybridization Core at Baylor College of Medicine using an automated robotic platform and procedures as described previously (Yaylaoglu et al., 2005) with minor modifications for double ISH. Briefly, fresh-frozen brains were embedded in optimal cutting temperature compound (OCT) and cryosectioned (25 µm). Two (*Stxbp1*/*Gad1* or *tdTomato/Gad1*) or three (*Stxbp1*/*Vglut1*/*Vglut2*) probes were hybridized to brain sections simultaneously in hybridization buffer (Ambion, catalog #B8807G). Sections were washed with standard saline citrate stringency solution (SSC; 0.15 M NaCl, 0.015 M sodium citrate) to remove unbound and nonspecifically bound probes. To visualize the DIG-labeled probe, we incubated brain sections for 30 min with a horse radish peroxidase (HRP)-conjugated sheep anti-DIG primary antibody (Sigma, catalog #11207733910) diluted at 1/500 in Tris-NaCl blocking buffer [TNB; 100 mM Tris, 150 mM NaCl, 0.5% (w/v) blocking reagent (PerkinElmer, catalog #FP1012), pH 7.6]. After washes in Tris-NaCl-Tween (TNT; 10 mM Tris-HCl, pH 8.0, 150 mM NaCl and 0.05% Tween 20) buffer, brain sections were then developed with tyramide-Cy3 Plus (Akoya Biosciences, catalog #NEL744001KT, 1/50 dilution in amplification diluent, 15 min). After washes in TNT buffer, the remaining HRP activity was quenched by a 10 min incubation in 0.2 M HCl. Sections were then washed in TNT, blocked in TNB for 15 min before incubation with an HRP-conjugated sheep anti-FITC antibody (Sigma, catalog #11426346910) diluted at 1/500 in TNB for 30 min. After washes in TNT, the FITC-labeled probe was visualized using tyramide-FITC Plus (Akoya Biosciences, catalog #NEL741001KT, 1/50 dilution in amplification diluent, 15 min). The slides were washed in TNT and stained with 4′,6-diamidino-2-phenylindole (DAPI; Invitrogen, catalog #D3571), washed again, removed from the machine, and mounted in ProLong Diamond (Invitrogen, catalog #P36961).

The brain sections of Viaat-cHet or Vglut2-cHet and their respective control mice were stained and imaged in parallel. Fluorescence images of brain sections were acquired using an Axio Zoom.V16 fluorescence microscope (Zeiss) and processed using Imaris (Oxford Instruments) or ImageJ (National Institutes of Health). The frontal cortex, somatosensory cortex, hippocampus, thalamus, reticular thalamic nucleus, striatum, and cerebellum were analyzed from the sagittal sections and the amygdala and hypothalamus from the coronal sections. Three to eight sections from each mouse were analyzed for each brain region. For *Gad1*- or *Vglut1/2*-positive cells, individual somas were selected using the surface function of Imaris with the following parameters: surface detail, 0.811; diameter of the largest square, 25 μm for cortical pyramidal neurons and Purkinje cells and 20 μm for other neurons; pixels with the intensity at the lower 2–4% range of the maximal intensity were removed; voxels with the size at the lower 1.5–2% range of the maximal voxel size were removed. The mean intensity of *Stxbp1* was measured in each of the selected somas, and then the average intensity was calculated across all selected cells for a brain section. For *Gad1-* or *Vglut1/2*-negative cells, *Gad1*- or *Vglut1/2*-positive cells were first selected as described above and removed. Individual *Gad1*- or *Vglut1/2*-negative somas were then selected based on *Stxbp1* signals using the parameters described above. The mean intensity of *Stxbp1* was measured in each of the selected somas, and the average intensity was calculated across all selected cells for a brain section. Approximately 50–100 *Gad1*-positive or *Vglut1/2*-negative cells and 200–600 *Vglut1/2*-positive or *Gad1*-negative cells were selected for a brain region except the striatum where ∼800 cells were selected in each section. For the hippocampal pyramidal neurons and cerebellar granular cells, due to their high cellular densities, the soma region of a group of cells instead of individual cells was selected manually, and the mean *Stxbp1* intensities were measured using ImageJ. Background signals were measured in intercellular space and subtracted from each measurement. *Stxbp1* levels from different brain sections were normalized by the average *Stxbp1* levels of WT brain sections that were simultaneously stained and imaged.

Tile scanned *z*-stack images of the sagittal sections of *Dlx5/6-Cre^Tg/+^;Rosa26^tdTomato/+^* mice were acquired on an Sp8X Confocal Microscope (Leica) using a 20× oil objective. Approximately 480 tiles were collected per tile scan with a 10% overlap between images and merged without smoothing. Each tile was composed of a *z*-stack centered at the optimum focal point with five optical sections taken with a *z*-step size of 1.51 µm for a total depth of 6.05 µm. Each tile was imaged at three wavelengths: DAPI (Ex, 405 nm; Em, 410–480 nm), FITC (Ex, 488 nm; Em, 497–535 nm), and Cy3 (Ex, 554 nm; Em, 564–580 nm). The entire *z*-stack was processed using the “Sum Slices” function in ImageJ (National Institutes of Health).

### Health monitoring

Body weight and hindlimb clasping of mice were monitored weekly. Hindlimb stiffness and clasping were assessed by holding mice on their tails briefly in the air and scoring the movement of hindlimbs as 0 = no stiffness or clasping, 1 = stiffness in hindlimb, 2 = clasping of one hindlimb, 3 = clasping of both hindlimbs, and 4 = tight clasping of both hindlimbs.

### Developmental milestones

Pinnae detachment, fur development, incision eruption, and eye opening were monitored from postnatal day (P) 0. Surface righting reflex and negative geotaxis reflex were evaluated on P3, 5, 7, 9, 11, and 13. The pups were tattooed at P3 for identification and genotyped after all tested were performed and analyzed. The sexes and ages of the tested mice were indicated in the figures. The details of mouse cohorts are reported in Extended Data Table 2-3. For surface righting reflex, a pup was placed gently on its back on a warm heated platform, and the amount of time for it to flip itself was recorded. If the pup did not flip within 60 s, then the test was stopped, and the amount of time was recorded as 60 s. The test was repeated three times on each test day. Negative geotaxis reflex test was performed after completing all three trials of surface righting reflex. A pup was placed on an inclined plane with its head facing downward, and the amount of time for it to orientate itself with its head facing upward was recorded. If the pup did not turn within 60 s, then the test was stopped, and the amount of time was recorded as 60 s. The test was repeated three times on each test day. The angle of incline was 20° for P3–7, 35° for P9–11, and 45° for P13.

### Behavioral tests

All behavioral experiments were performed using the equipment and facility at the Neurobehavioral Core of Baylor College of Medicine Intellectual and Developmental Disabilities Research Center. Behavioral tests were performed and analyzed blind to the genotypes according to the protocols published previously (Chen et al., 2020) with minor modifications. Four sex- and age-matched WT, Flox, Cre, and cHet littermate were housed together in one cage. Approximately equal numbers of cHet mice and their sex- and age-matched WT, Flox, and Cre littermates were tested in parallel in each experiment except for resident–intruder test where only male mice were used. Mice were habituated in the behavioral test facility for at least 30 min before testing. The sexes and ages of the tested mice were indicated in the figures. The details of mouse cohorts and performed tests are reported in Extended Data Table 2-3.

#### Nesting test

An autoclaved Nestlet was given to a mouse individually housed in its home cage, and the quality of the nest was scored after 24 h.

#### Elevated plus maze test

A mouse was placed in the center of an elevated maze consisting of two open arms (25 × 8 cm) and two closed arms with high walls (25 × 8 × 15 cm). The mouse was initially placed facing the open arms and then allowed to freely explore for 10 min with 150–200 lux illumination and 65 dB background white noise. The mouse activity was recorded using a video camera (ANY-maze, Stoelting).

#### Open-field test

A mouse was placed at the center of a clear, open chamber (40 × 40 × 30 cm) and allowed to freely explore for 30 min with 150–200 lux illumination and 65 dB background white noise. The horizontal plane was evenly divided into 256 squares (16 × 16), and the center zone is defined as the central 100 squares (10 × 10). The horizontal travel and vertical activity were quantified by either an Open Field Locomotor system or a VersaMax system (OmniTech).

#### Marble burying test

A clean standard housing cage was filled with ∼8 cm deep bedding material. Twenty marbles were arranged on top of the bedding in a 4 × 5 array. A mouse was placed into this cage for 30 min before the number of buried marbles (i.e., at least 50% of the marble covered by the bedding material) was recorded.

#### Rotarod test

A mouse was tested on an accelerating rotarod apparatus (Ugo Basile) in three trials per day for 4 consecutive days. There was a 30–60 min resting interval between trials. Each trial lasted for a maximum of 5 min, during which the rod accelerated linearly from 4 to 40 revolutions per minute (rpm). The time when the mouse walks on the rod and the latency for the mouse to fall from the rod were recorded for each trial.

#### Foot slip test

A mouse was placed onto an elevated 40 × 25 cm wire grid (1 × 1 cm spacing) and allowed to freely move for 5 min. The number of foot slips was manually counted, and the moving distance was measured through a video camera (ANY-maze, Stoelting). The number of foot slips was normalized by the moving distance for each mouse.

#### Vertical pole test

A mouse was placed at the top of a vertical threaded metal pole (1.3 cm diameter, 55 cm length). The amount of time for the mouse to descend to the floor was measured with a maximal cutoff time of 120 s.

#### Hole-board test

A mouse was placed at the center of a clear chamber (40 × 40 × 30 cm) that contains a floor with 16 evenly spaced holes (5/8 inch diameter) arranged in a 4 × 4 array. The mouse was allowed to freely explore for 10 min. Its open-field activity above the floorboard and nose pokes into the holes were detected by infrared beams above and below the hole board, respectively, using the VersaMax system (OmniTech).

#### Acoustic startle response test

A mouse was placed in a plastic cylinder and acclimated to the 70 dB background white noise for 5 min. The mouse was then tested with four blocks, and one block consisted of 13 trials. In one block, each of 13 different levels of sound (70, 74, 78, 82, 86, 90, 94, 98, 102, 106, 110, 114, or 118 dB, 40 ms, intertrial interval of 15 s on average) was presented in a pseudorandom order. The startle response was recorded for 40 ms after the onset of the sound. The rapid force changes due to the startles were measured by an accelerometer (SR-LAB, San Diego Instruments).

#### Prepulse inhibition test

A mouse was placed in a plastic cylinder and acclimated to the 70 dB background noise for 5 min. The mouse was then tested with six blocks, and one block consisted of eight trials in a pseudorandom order: a “no stimulus” trial (40 ms, only 70 dB background noise present), a test pulse trial (40 ms, 120 dB), three different prepulse trials (20 ms, 74, 78, or 82 dB), and three different prepulse inhibition trials (a 20 ms, 74, 78, or 82 dB prepulse preceding a 40 ms, 120 dB test pulse by 100 ms). The startle response was recorded for 40 ms after the onset of the 120 dB test pulse. The intertrial interval is 15 s on average. The rapid force changes because the startles were measured by an accelerometer (SR-LAB, San Diego Instruments). Prepulse inhibition of the startle responses was calculated as “1 – (prepulse inhibition trial/test pulse trial)”.

#### Hot plate test

A mouse was placed on a hot plate (Columbus Instruments) with a temperature of 55°C. The latency for the mouse to first respond with either a hindpaw lick, hindpaw flick, or jump was recorded. If the mouse did not respond within 45 s, then the test was terminated, and the latency was recorded as 45 s.

#### Three-chamber test

The apparatus (60.8 × 40.5 × 23 cm) consists of three chambers (left, center, and right) of equal size with 10 × 5 cm openings between the chambers. A test mouse was placed in the apparatus with a mesh pencil cup in each of the left and right chambers and allowed to freely explore for 10 min. A novel object was then placed under one mesh pencil cup and an age- and sex-matched partner mouse (WT C57BL/6J) under the other mesh pencil cup. The test mouse was allowed to freely explore for another 10 min. The position of the test mouse was tracked through a video camera (ANY-maze, Stoelting), and the approaches of the test mouse to the object or partner mouse were scored manually. Partner mice were habituated to the mesh pencil cups in the apparatus for 1 h per day for 2 d prior to testing. A partner mouse was used only in one test per day.

#### Resident–intruder test

Male test mice (resident mice) were individually caged for 2 weeks before testing. Age-matched male BALB/cAnNTac or BALB/cJ mice were group-housed to serve as the intruders. During the test, an intruder was placed into the home cage of a test mouse for 10 min, and their behaviors were video recorded. Videos were scored for the number and duration of each attack by the resident mouse regardless the attack was initiated by either the resident or intruder.

#### Novel object recognition test

A mouse was first habituated in an empty arena (24 × 45 × 20 cm) for 5 min before every trial and then placed into the testing arena with two identical objects (i.e., familiar object 1 and familiar object 2) for the first three trials. In the fourth trial, familiar object 1 was replaced with a novel object. In the fifth trial, the mouse was presented with the two original, identical objects again. Each trial lasted 5 min. The intertrial interval was 24 h. The movement of mice was recorded by a video camera. The amount of time that the mouse interacted with the objects (*T*) was recorded using a wireless keyboard (ANY-maze, Stoelting). The preference index of interaction was calculated as *T*_familiar object 1_* / *(*T*_familiar object 1_* + T*_familiar object 2_) for the first three trials and fifth trial and as *T*_novel object_* / *(*T*_novel object_* + T*_familiar object 2_) for the fourth trial.

#### Fear conditioning test

Pavlovian fear conditioning was conducted in a chamber (30 × 25 × 29 cm) with a grid floor for delivering electrical shocks (Coulbourn Instruments). During the 5 min training phase, a mouse was placed in the chamber for 2 min, and then a sound (85 dB, white noise) was turned on for 30 s immediately followed by a mild footshock (2 s, 0.72 mA). The same sound and footshock were repeated one more time 2 min after the first footshock. After the second footshock, the mouse stayed in the training chamber for 18 s before returning to its home cage. After 24 h, the mouse was tested for the contextual and cued fear memories. In the contextual fear test, the mouse was placed in the same training chamber, and its freezing behavior was monitored for 5 min without the sound stimulus. The mouse was then returned to its home cage. One to 2 h later, the mouse was transferred to the chamber after it has been altered using plexiglass inserts and a different odor to create a new context for the cued fear test. After 3min in the chamber, the same sound cue that was used in the training phase was turned on for 3 min without footshocks while the freezing behavior was monitored. The freezing behavior was scored using an automated video-based system (FreezeFrame, Actimetrics). The freezing time (%) during the first 2 min of the training phase (i.e., before the first sound) was subtracted from the freezing time (%) during the contextual fear test to obtain context-induced freezing time. The freezing time (%) during the first 3 min of the cued fear test (i.e., without sound) was subtracted from the freezing time (%) during the last 3 min of the cued fear test (i.e., with sound) to obtain the cue-induced freezing time.

### Video-EEG/EMG

Video-electroencephalography and electromyography (EEG/EMG) recordings and analysis were performed as previously described (Chen et al., 2020). Briefly, mice at 8–13 weeks of age were anesthetized with 2.5% isoflurane in oxygen. Approximately 0.25-mm-diameter craniotomies were performed at the coordinates below that were normalized by the distance between bregma and lambda (DBL). Perfluoroalkoxy polymer (PFA)-coated silver wire electrodes (A-M Systems, catalog #786000, 127 mm bare diameter, 177.8 mm coated diameter) were used for grounding at the right frontal cortex, referencing at the cerebellum, and recording at the left frontal cortex [anterior posterior (AP), 0.42 of DBL; medial lateral (ML), 0.356 of DBL; dorsal ventral (DV), −1.5 mm], left, and right somatosensory cortices (AP, −0.34 of DBL; ML, ±0.653 of DBL; DV, −1.5 mm). An EMG recording and an EMG reference electrode were inserted into the neck muscles. The mice were allowed to recover from the surgeries for at least 1 week. Before recording, mice were individually habituated in the recording chambers for 24 h. EEG/EMG signals (5,000 Hz sampling rate with a 0.5 Hz high-pass filter) and videos (30 frames per second) were recorded synchronously for continuous 72 h using a four-channel EEG/EMG tethered system (Pinnacle Technology).

SWDs were identified by generating putative candidates with custom-written code in MATLAB (MathWorks) followed by the classification of candidates with a convolutional neural network in Python that has been trained with manually labeled EEG segments (Chen et al., 2020). Myoclonic seizures were identified by visual inspection of EEG/EMG signals and videos to identify sudden jumps and jerks (Chen et al., 2020). The state of the mouse before each myoclonic seizure event was classified as rapid eye movement (REM) sleep, nonrapid eye movement (NREM) sleep, or awake based on the EEG/EMG. The video component of the data file for one Vglut2-cHet mouse was corrupted, precluding the identification of myoclonic seizures. Thus, this mouse was only analyzed for SWDs.

### Experimental design and statistical analysis

The sample sizes were estimated by G*Power 3 program (Faul et al., 2007) based on pilot experiments and the previous study (Chen et al., 2020) that used similar assays. They are also within the range that is generally accepted in the field. All experiments were performed and analyzed blind to the genotypes. Approximately equal number of male and female mice was included in experiments. No data point was excluded. All reported sample numbers (*n*) represent independent biological replicates that are the numbers of tested mice or tissue sections (see below). Statistical analyses were performed with Prism 9 (GraphPad software) unless stated otherwise. Anderson–Darling test, D’Agostino–Pearson’s, Shapiro–Wilk, and Kolmogorov–Smirnov tests were used to determine if behavior and EEG data were normally distributed. If all data within one experiment passed all four normality tests, then the statistical test that assumes a Gaussian distribution was used. Otherwise, the statistical test that assumes a non-Gaussian distribution was used. Nested one-way ANOVA with Tukey’s multiple comparison was used to assess statistical significance of *Stxbp1* expression levels in the DFISH experiments. Either one-way or two-way ANOVA with multiple comparison was used for behavior and EEG data analyses. For data with Gaussian distribution, ordinary one-way ANOVA with Tukey's multiple comparison was used. For non-Gaussian distributed data, Kruskal–Wallis one-way ANOVA with Dunn's multiple comparison was used. Tukey’s multiple comparison was also used in conjunction with two-way ANOVA. For Western blot data, ordinary one-way ANOVA with Tukey’s multiple comparison was used. For body weight, hindlimb clasping score, rotarod test, acoustic startle response, and novel object recognition, two-way repeated-measures ANOVA was used with Tukey’s multiple comparison. Either two-way or three-way ANOVA with Tukey’s multiple comparison was used for sex effect analyses. OriginPro 2021 (OriginLab) was used to perform three-way ANOVA. To compare the difference between the hindlimb clasping scores of Viaat-cHet and Vglut2-cHet mice, we performed Fisher–Freeman–Halton exact test using StatXact 12 (Cytel). The details of all statistical tests, numbers of replicates, and *p* values are reported in Extended Data Table 1-7.

## Results

### Generation of a new *Stxbp1* flox allele in mice

**Figure 1.**
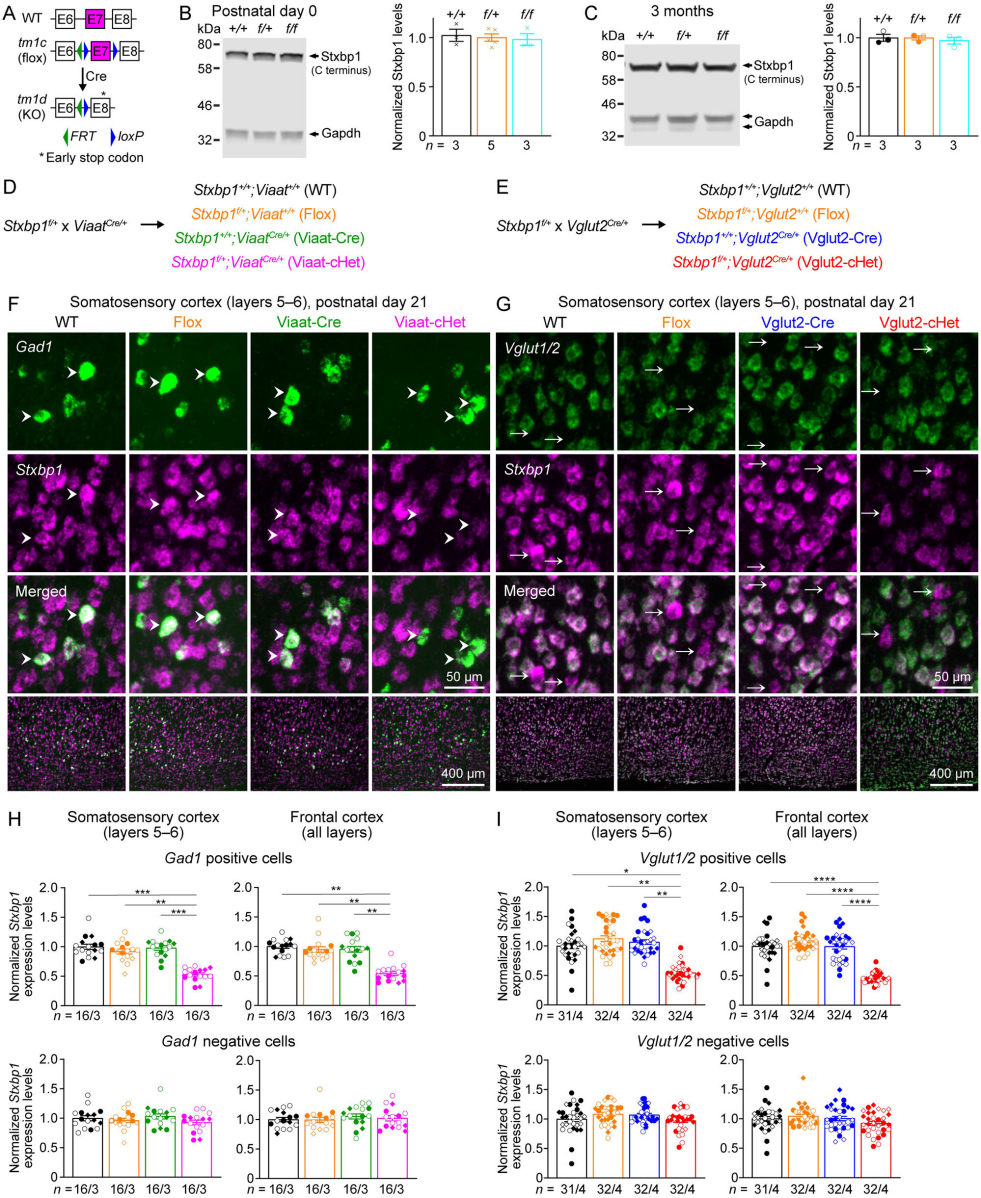
Generation and validation of Viaat-cHet and Vglut2-cHet mice. ***A***, Genomic structures of *Stxbp1* WT, *tm1c* (flox), and *tm1d* (KO) alleles. In the flox allele, exon 7 is flanked by two *loxP* sites. Cre-mediated recombination in the flox allele deletes exon 7, resulting in the KO allele with an early stop codon in exon 8. E, exon; *FRT*, Flp recombination site; *loxP*, Cre recombination site. ***B***, Left, A representative Western blot of proteins from the brains of WT, *Stxbp1^f/+^*, and *Stxbp1^f/f^* mice at P0. Stxbp1 was detected by an antibody recognizing its C terminus. Gapdh, a housekeeping protein as loading control. Right, summary data of normalized Stxbp1 protein levels at P0. Stxbp1 levels were first normalized by the Gapdh levels and then by the average Stxbp1 levels of all WT mice from the same blot. Each cross represents one mouse. ***C***, Similar to ***B***, but for the cortices of 3-month-old WT, *Stxbp1^f/+^*, and *Stxbp1^f/f^* mice. Each filled (male) or open (female) circle represents one mouse. ***D***, ***E***, *Stxbp1^f/+^* mice were crossed to *Viaat^Cre/+^* (***D***) or *Vglut2^Cre/+^* (***E***) mice to generate different genotypes of mice for experiments. The color scheme is maintained across all figures. ***F***, ***G***, Representative fluorescence images from brain sections labeled by ISH probes against *Stxbp1* and *Vglut1/2* (***F***) or *Gad1* (***G***) at P21. The sequences of the probes are provided in Extended Data List 1-1. The bottom row shows the layers 5–6 of the somatosensory cortex, and the top 3 rows show the individual cells from this region. Arrow heads (***F***) indicate *Gad1*-positive cells, and arrows (***G***) indicate *Vglut1/2*-negative cells. ***H***, Summary data of normalized *Stxbp1* mRNA levels in *Gad1*-positive (top row) and *Gad1*-negative (bottom row) cells from the somatosensory and frontal cortices. *Stxbp1* levels were normalized by the average *Stxbp1* levels of WT brain sections that were stained and imaged in parallel. The *Stxbp1* levels of *Gad1*-positive, but not *Gad1*-negative, cells in Viaat-cHet mice were reduced. Different shapes of symbols represent different mice (3 mice per genotype, filled symbols for males and open symbols for females), and each symbol represents one brain section. The DFISH results from other brain regions are provided in Extended Data Figure 1-2. Stxbp1 protein levels are shown in Extended Data Figure 1-3. ***I***, Similar to ***H***, but for *Vglut1/2*-positive and *Vglut1/2*-negative cells in Vglut2-cHet and control mice and four mice per genotype. The DFISH results from other brain regions are provided in Extended Data Figure 1-4. The Stxbp1 protein levels are shown in Extended Data Figure 1-5. The Viaat and Vglut2 protein levels are shown in Extended Data Figure 1-6. Data are mean ± SEM. *p< 0.05, **p < 0.01, ***p < 0.001, ****p < 0.0001 (see the details of all statistical tests in Extended Data Table 1-7).

We crossed a previously generated *Stxbp1^tm1a/+^* mouse (Chen et al., 2020) with a Flp recombinase germline deleter mouse to create a new *Stxbp1* flox allele (*tm1c*), in which exon 7 is flanked by two *loxP* sites (Fig. 1*A*). Heterozygous (*Stxbp1^f/+^*) and homozygous (*Stxbp1^f/f^*) flox mice are viable and fertile. Western blots showed that *Stxbp1^f/+^* and *Stxbp1^f/f^* mice had similar Stxbp1 protein levels to their WT littermates at P0 and 3 months of age (Fig. 1*B*,*C*), indicating that the presence of *FRT* and *loxP* sites does not affect Stxbp1 expression. Deletion of exon 7 by Cre recombinase will lead to an early stop codon in exon 8 (Fig. 1*A*), resulting in the same *Stxbp1 tm1d* null allele described previously (Chen et al., 2020).

10.1523/JNEUROSCI.1806-23.2024.f1-2 Extended Data Figure 1-2 **Reduction of *Stxbp1* mRNA levels specifically in GABAergic neurons of Viaat-cHet mice.** (**A**) Representative fluorescence images from brain sections labeled by ISH probes against *Stxbp1* and *Gad1*. The bottom row shows the layers 1–4 of the somatosensory cortex, and the top three rows show the individual cells from this region. Arrows heads indicate *Gad1*-positive cells. (**B–I**) Similar to (A), but for other brain regions indicated on the top of each panel. TRN, thalamic reticular nucleus; LHA, lateral hypothalamic area; CP, caudoputamen. (**J**) Summary data of normalized *Stxbp1* mRNA levels in *Gad1­*-positive cells from different brain regions. *Stxbp1* levels were normalized by the average *Stxbp1* levels of WT brain sections that were stained and imaged in parallel. The *Stxbp1* levels of Viaat-cHet mice were reduced in all brain regions. Different shapes of symbols represent different mice (3 mice per genotype, filled circles for 1 male and open circles and diamonds for 2 females), and each symbol represents one brain section. (**K**) Similar to (J), but for *Gad1­*-negative cells from different brain regions. The *Stxbp1* levels of *Gad1­*-negative cells in Viaat-cHet mice were normal. Data are mean ± s.e.m. * *P* < 0.05, ** *P* < 0.01, *** *P* < 0.001, **** *P* < 0.0001. Download Extended Data Figure 1-2, TIF file.

10.1523/JNEUROSCI.1806-23.2024.f1-3 Extended Data Figure 1-3 **Mild reduction of Stxbp1 protein levels in Viaat-cHet mice.** (**A**) Representative Western blots of proteins from different brain regions of WT, Flox, Viaat-Cre, and Viaat-cHet mice at the age of 11–19 weeks. Stxbp1 was detected by an antibody recognizing its N terminus. Gapdh, a housekeeping protein as loading control. (**B**) Summary data of normalized Stxbp1 protein levels. Stxbp1 levels were first normalized by the Gapdh levels and then by the average Stxbp1 levels of all WT mice from the same blot. Each filled (male) or open (female) circle represents one mouse. Data are mean ± s.e.m. * *P* < 0.05, ** *P* < 0.01. Download Extended Data Figure 1-3, TIF file.

10.1523/JNEUROSCI.1806-23.2024.f1-4 Extended Data Figure 1-4 **Reduction of *Stxbp1* mRNA levels specifically in glutamatergic neurons of Vglut2-cHet mice.** (**A**) Representative fluorescence images from brain sections labeled by ISH probes against *Stxbp1* and *Vglut1/2*. The bottom row shows the layers 1–4 of the somatosensory cortex, and the top three rows show the individual cells from this region. Arrows indicate *Vglut1/2*-negative cells. (**B–I**) Similar to (A), but for other brain regions indicated on the top of each panel. VMH, ventromedial hypothalamic nucleus; TRN, thalamic reticular nucleus; CP, caudoputamen. (**J**) Summary data of normalized *Stxbp1* mRNA levels in *Vglut1/2­*-positive cells from different brain regions. *Stxbp1* levels were normalized by the average *Stxbp1* levels of WT brain sections that were stained and imaged in parallel. The *Stxbp1* levels of Vglut2-cHet mice were reduced in most brain regions except cerebellar granule cells. Different shapes of symbols represent different mice (4 mice per genotype, filled circles and diamonds for 2 males and open circles and diamonds for 2 females), and each symbol represents one brain section. (**K**) Similar to (J), but for *Vglut1/2­*-negative cells from different brain regions. The *Stxbp1* levels of *Vglut1/2­*-negative cells in Vglut2-cHet mice were normal. Data are mean ± s.e.m. * *P* < 0.05, ** *P* < 0.01, *** *P* < 0.001, **** *P* < 0.0001. Download Extended Data Figure 1-4, TIF file.

10.1523/JNEUROSCI.1806-23.2024.f1-5 Extended Data Figure 1-5 **Reduction of Stxbp1 protein levels in Vglut2-cHet mice.** (**A**) Representative Western blots of proteins from different brain regions of WT, Flox, Vglut2-Cre, and Vglut2-cHet mice at the age of 8–9 weeks. Stxbp1 was detected by an antibody recognizing its N terminus. Gapdh, a housekeeping protein as loading control. (**B**) Summary data of normalized Stxbp1 protein levels. Stxbp1 levels were first normalized by the Gapdh levels and then by the average Stxbp1 levels of all WT mice from the same blot. Each filled (male) or open (female) circle represents one mouse. Data are mean ± s.e.m. * *P* < 0.05, ** *P* < 0.01, *** *P* < 0.001, **** *P* < 0.0001. Download Extended Data Figure 1-5, TIF file.

10.1523/JNEUROSCI.1806-23.2024.f1-6 Extended Data Figure 1-6 **Normal Viaat and Vglut2 protein levels in Viaat-cHet and Vglut2-cHet mice, respectively.** (**A**) Representative Western blots of Viaat proteins from different brain regions of WT, Flox, Viaat-Cre, and Viaat-cHet mice at the age of 11–19 weeks. Gapdh, a housekeeping protein as loading control. (**B**) Summary data of normalized Viaat protein levels. Viaat levels were first normalized by the Gapdh levels and then by the average Viaat levels of all WT mice from the same blot. (**C,D**) Similar to (A,B), but for Vglut2 protein levels in WT, Flox, Vglut2-Cre, and Vglut2-cHet mice at the age of 8–9 weeks. Each filled (male) or open (female) circle represents one mouse. Data are mean ± s.e.m. * *P* < 0.05. Download Extended Data Figure 1-6, TIF file.

10.1523/JNEUROSCI.1806-23.2024.t1-7 Extended Data Table 1-7 **Statistics of experimental results.** The details of all statistical tests, numbers of replicates, and *P* values are presented for each experiment in the table. Download Extended Data Table 1-7, XLSX file.

### Conditional *Stxbp1* haploinsufficiency in GABAergic/glycinergic or glutamatergic neurons

To create *Stxbp1* haploinsufficiency broadly in GABAergic/glycinergic neurons, we used a well-characterized *Viaat-ires-Cre* line (Vong et al., 2011) because Viaat (also called Slc32a1) is the only vesicular transporter loading both inhibitory neurotransmitters GABA and glycine into synaptic vesicles and is required for presynaptic transmitter release in both GABAergic and glycinergic neurons (Wojcik et al., 2006; Rahman et al., 2015). Allen Brain Atlas used a Cre-dependent tdTomato reporter line Ai14, *Rosa26-CAG-LSL-tdTomato* (Madisen et al., 2010) to characterize the expression pattern of *Viaat-ires-Cre* mice by DFISH with probes against *tdTomato* and *Gad1* (*glutamate decarboxylase 1* that labels GABAergic neurons). In *Viaat^Cre/+^;Rosa26^tdTomato/+^* mice, *tdTomato* is expressed in *Gad1*-positive cells throughout the brain (https://connectivity.brain-map.org/transgenic/experiment/100142488).

To create *Stxbp1* haploinsufficiency broadly in glutamatergic neurons, we used *vesicular glutamate transporter 2* (*Vglut2)-ires-Cre* (Vong et al., 2011) because Vglut2 (also called Slc17a6) is expressed in all glutamatergic neurons at embryonic and early postnatal stages, and Cre-mediated recombination can occur in all glutamatergic neurons (Boulland et al., 2004). The expression pattern of *Vglut2-ires-Cre* mice was also characterized by Allen Brain Atlas using *Rosa26-CAG-LSL-tdTomato* and DFISH with probes against *tdTomato* and *Gad1*. Indeed, *tdTomato* is broadly expressed in *Gad1*-negative cells throughout the brain including those areas where Vglut2 expression is downregulated in adult mice (e.g., the cortex, hippocampus, and cerebellum; https://connectivity.brain-map.org/transgenic/experiment/100142487).

We then crossed *Stxbp1^f/+^* mice with *Viaat^Cre/+^* mice to obtain four genotypes: *Stxbp1^+/+^;Viaat^+/+^* (WT), *Stxbp1^f/+^;Viaat^+/+^* (Flox), *Stxbp1^+/+^;Viaat^Cre/+^* (Viaat-Cre), and *Stxbp1^f/+^;Viaat^Cre/+^* (Viaat-cHet; Fig. 1*D*). Similarly, *Stxbp1^f/+^* mice were crossed with *Vglut2^Cre/+^* mice to obtain *Stxbp1^+/+^;Vglut2^+/+^* (WT), *Stxbp1^f/+^;Vglut2^+/+^* (Flox), *Stxbp1^+/+^;Vglut2^Cre/+^* (Vglut2-Cre), and *Stxbp1^f/+^;Vglut2^Cre/+^* (Vglut2-cHet; Fig. 1*E*). To validate Cre-mediated conditional deletion of one *Stxbp1* allele in Viaat-cHet and Vglut2-cHet mice, we used DFISH to examine *Stxbp1* mRNA levels in specific neurons because Stxbp1 is present throughout the cells (Ramos-Miguel et al., 2015), and we found that immunostaining of Stxbp1 in brain sections could not precisely and reliably differentiate the signals in one neuron from those in the neurites of neighboring neurons. Since a successful Cre-mediated recombination will convert the *tm1c* allele to *tm1d*, and the *tm1d* transcripts will be degraded due to nonsense-mediated decay (Chen et al., 2020), DFISH allows us to determine the specificity and efficiency of recombination in specific cell types. We examined both glutamatergic and GABAergic neurons in major brain regions including the cortex, hippocampus, thalamus, striatum, amygdala, hypothalamus, and cerebellum.

For Viaat-cHet mice, we used *Gad1* probe to label GABAergic neurons. *Stxbp1* mRNA levels in Viaat-cHet were reduced by 36–59% in GABAergic neurons of all examined brain regions (Fig. 1*F*,*H*; Extended Data Fig. 1-2). These results showed that *Viaat-ires-Cre* broadly and efficiently deleted one *Stxbp1* allele in GABAergic neurons. In contrast, *Stxbp1* mRNA levels in the *Gad1*-negative neurons of the cortex, thalamus, and cerebellum, which are vastly glutamatergic neurons, were unaltered (Fig. 1*F*,*H*; Extended Data Fig. 1-2), confirming the specificity of *Viaat-ires-Cre*. We also performed Western blot analyses on proteins extracted from different brain regions and found that compared with three control groups (WT, Flox, and Viaat-Cre), Stxbp1 was slightly reduced (i.e., <24%) in most brain regions of Viaat-cHet mice (Extended Data Fig. 1-3), consistent with GABAergic/glycinergic cells being a small fraction of neurons. Even though most striatal neurons are GABAergic cells, the reduction of Stxbp1 in the striatum was also modest, most likely because the striatum receives numerous glutamatergic inputs from other brain regions, which constitute ∼80% of the synapses in the striatum (Wilson, 2007), and these synapses and axons should contain normal levels of Stxbp1 (see below). Since *Viaat-ires-Cre* also efficiently mediates recombination in glycinergic neurons (Ausborn et al., 2018), Viaat-cHet mice are indeed GABAergic/glycinergic neuron-specific *Stxbp1* haploinsufficient mice.

For Vglut2-cHet mice, we combined the probes against *Vglut1* and *Vglut2* to label all glutamatergic neurons with a single color. Compared with the three control groups (WT, Flox, and Vglut2-Cre), *Stxbp1* mRNA levels in Vglut2-cHet were reduced by 44–60% in the glutamatergic neurons of examined brain regions except cerebellar granular cells where the reduction was ∼15–21% (Fig. 1*G*,*I*; Extended Data Fig. 1-4). The modest reduction of *Stxbp1* mRNA levels in cerebellar granular cells is consistent with a similar degree of reduction of Stxbp1 protein levels in the cerebellum of *Stxbp1^tm1d/+^* (Chen et al., 2020) and Vglut2-cHet mice (see below). These results showed that *Vglut2-ires-Cre* broadly and efficiently deleted one *Stxbp1* allele in glutamatergic neurons. In contrast, *Stxbp1* mRNA levels in the *Vglut1/Vglut2*-negative neurons of the cortex, thalamic reticular nucleus, and striatum, which are vastly GABAergic neurons, were unaltered (Fig. 1*G*,*I*; Extended Data Fig. 1-4), confirming the specificity of *Vglut2-ires-Cre*. Western blot analyses showed that Stxbp1 was significantly reduced in all examined brain regions of Vglut2-cHet mice including the striatum that contains GABAergic cells and glutamatergic synapses from other brain regions (Wilson, 2007; Extended Data Fig. 1-5).

We also determined the Viaat and Vglut2 protein levels of several brain regions where they are highly expressed in Viaat-cHet and Vglut2-cHet mice, respectively, because the insertion of the *ires-Cre* cassette after the stop codon of endogenous genes may reduce the gene expression (Viollet et al., 2017; Cheng et al., 2019; Joye et al., 2020; Straub et al., 2020). Western blot analyses showed neither significant changes of Viaat in Viaat-cHet mice nor Vglut2 in Vglut2-cHet mice as compared with their control mice (Extended Data Fig. 1-6).

Guided by the phenotypes of constitutive haploinsufficient mice *Stxbp1^tm1d/+^* and *Stxbp1^tm1a/+^* (Chen et al., 2020) and symptoms of *STXBP1* encephalopathy patients, we sought to characterize the neurological functions of male and female Viaat-cHet and Vglut2-cHet mice in comparison with their sex- and age-matched control littermates to dissect the contributions of GABAergic/glycinergic and glutamatergic neurons to *STXBP1* encephalopathy pathogenesis (see Extended Data Table 2-3 for the details of mouse cohorts used in the behavioral tests). The mouse genetic backgrounds, insertion of *loxP* sites, insertion of *Cre* cassettes, or Cre expression itself can all potentially affect the phenotypes. Therefore, we only concluded that *Stxbp1* haploinsufficiency in GABAergic/glycinergic or glutamatergic neurons significantly altered a phenotype if Viaat-cHet or Vglut2-cHet mice, respectively, were statistically different from at least both Flox and Cre control mice. We included WT mice as a control group to evaluate if Flox or Cre mice had any phenotypes.

### *Stxbp1* haploinsufficiency in GABAergic/glycinergic neurons causes reduced survival, developmental delay, hindlimb clasping, and impaired nesting behavior

**Figure 2.**
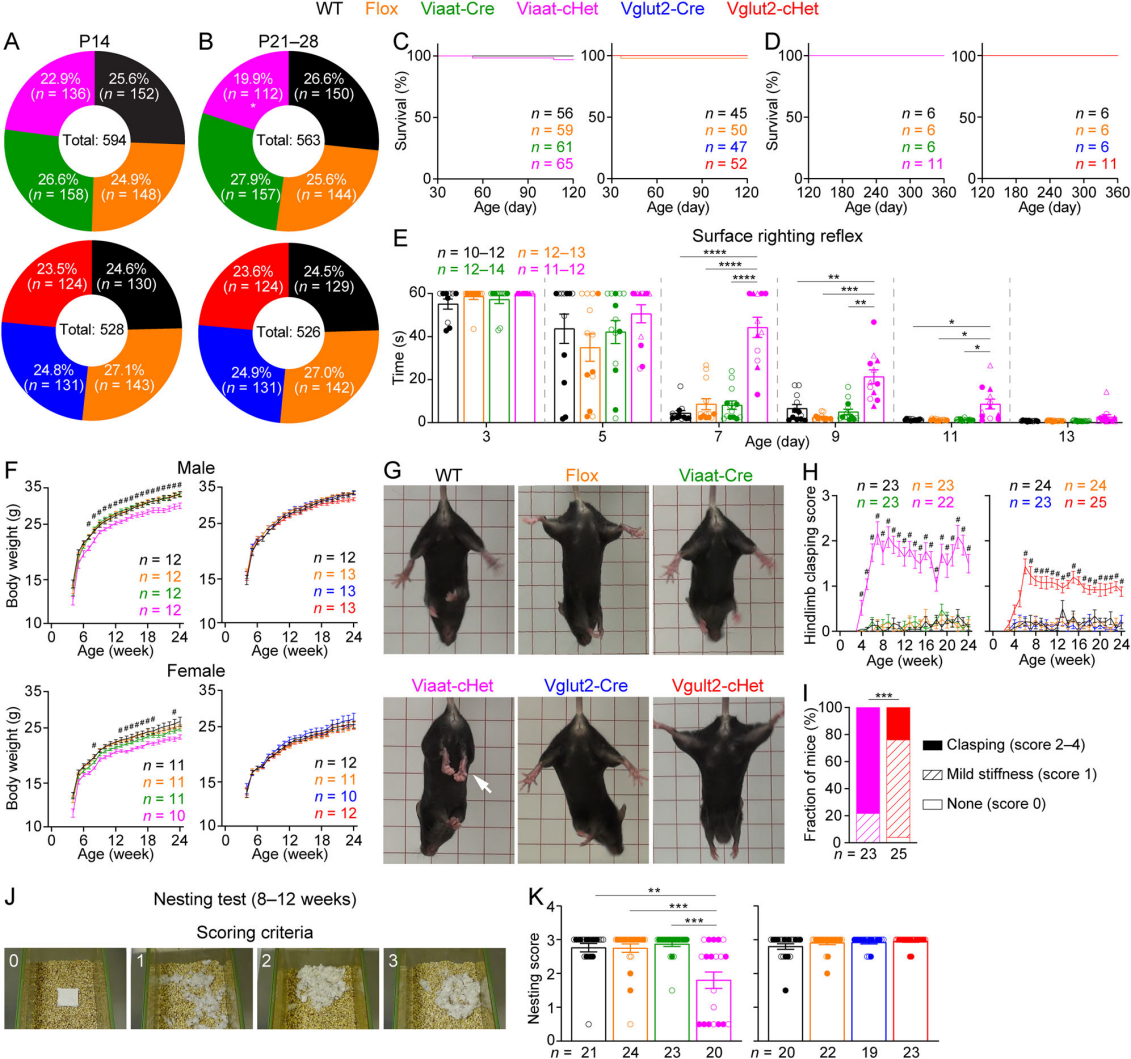
Early lethality, developmental delay, hindlimb clasping, and impaired nesting behavior of Viaat-cHet mice. ***A***, ***B***, Pie charts showing the observed numbers of mice with different genotypes at P14 (***A***) and P21–28 (***B***). The total numbers of observed mice are shown in the middle. Viaat-cHet mice were significantly less than Mendelian expectations at P21–28. ***C***, ***D***, Both Viaat-cHet and Vglut2-cHet mice had normal survival rates after P30. Note, only a subset of mice were observed up to P360 for the survival analysis (***D***). ***E***, The amount of time it took for the pup to flip onto its feet from a supine position as a function of age. The maturation of this surface righting reflex was delayed in Viaat-cHet mice. Note, the filled (male) and open (female) triangles represent those pups that later died between P14 and P21. The results of other developmental milestones are shown in Extended Data Figure 2-1. The cohorts of mice used in the developmental milestone and behavioral experiments are provided in Extended Data Table 2-2. ***F***, Body weight as a function of age. The body weight of Viaat-cHet mice was less than that of control mice. ^#^indicates that Viaat-cHet mice are statistically different (i.e., at least *p* < 0.05) from at least both Flox and Viaat-Cre mice. ***G***, Viaat-cHet mice showed hindlimb clasping (arrows), and Vglut2-cHet mice showed mild stiffness (Extended Data Video 2-3). ***H***, Hindlimb clasping scores as a function of age. ^#^ indicates that Viaat-cHet and Vglut2-cHet mice are statistically different (i.e., at least *p* < 0.05) from at least both corresponding Flox and Cre mice. ***I***, The fractions of Viaat-cHet and Vglut2-cHet mice with different severities of hindlimb stiffness or clasping. ***J***, ***K***, The quality of the nests was scored according to the criteria in ***J***. Viaat-cHet mice built poor quality nests within 24 h. For different panels, the numbers and ages of tested mice are indicated in the figure. Each filled (male) or open (female) circle represents one mouse. Data in (***E***, ***F***, ***H***, ***K***) are mean ± SEM. *p < 0.05, **p < 0.01, ***p < 0.001, ****p < 0.0001.

Viaat-cHet mice were born at the expected Mendelian frequency (24% of 51 pups; *p* = 0.98) and were also observed at the expected Mendelian frequency at P14 (Fig. 2*A*). However, ∼20% of Viaat-cHet mice died between P14 and P28, resulting in less Viaat-cHet mice than the Mendelian expectation (Fig. 2*B*). Interestingly, those Viaat-cHet mice that survived through this period had a similar survival rate as the control mice when monitored up to 1 year (Fig. 2*C*,*D*). This partially penetrant postnatal lethality phenotype of Viaat-cHet mice is similar to that of *Stxbp1^tm1d/+^* and *Stxbp1^tm1a/+^* mice (Chen et al., 2020), but its underlying cause is currently unknown. In contrast, Vglut2-cHet mice had a normal survival rate (Fig. 2*A–D*).

10.1523/JNEUROSCI.1806-23.2024.f2-1 Extended Data Figure 2-1 **Developmental milestones of Viaat-cHet mice.** (**A**) The postnatal days when the developmental milestones including pinnae detachment, fur development, incisor eruption, and eye opening were first observed. Note, due to illness of the experimenter, the eye opening days of a subset of mice were not recorded. (**B**) When placing on an inclined plane with the head facing downwards, the amount of time it took for the pup to orientate itself with its head facing upwards as a function of age. The negative geotaxis reflex was normal in Viaat-cHet mice. Note, the filled (male) and open (female) triangles represent those pups that later died between P14–21. (**C**) Body weight as a function of age. The body weight of Viaat-cHet mice was not significantly different from that of control mice during the first 3 postnatal weeks. For different panels, the numbers and ages of tested mice are indicated in the figures. Each filled (male) or open (female) circle represents one mouse. Data are mean ± s.e.m. * *P* < 0.05. Download Extended Data Figure 2-1, TIF file.

10.1523/JNEUROSCI.1806-23.2024.t2-2 Extended Data Table 2-2 **Mouse cohorts for behavioral experiments.** The details of all cohorts of mice used in the developmental milestone and behavioral tests are provided. Download Extended Data Table 2-2, XLSX file.

10.1523/JNEUROSCI.1806-23.2024.video.2-3 Extended Data Video 2-3 **Hindlimb clasping or stiffness in Viaat-cHet and Vglut2-cHet mice.** Representative videos showing the movements of WT, Flox, Viaat-Cre, Viaat-cHet, Vglut2-Cre, and Vglut2-cHet mice when they were picked up by the tails. Note, the hindlimbs of the Viaat-cHet mouse were stiff and often clasped, whereas those of the control mice were flexible. The hindlimbs of the Vglut2-cHet mouse only showed mild stiffness. Download Extended Data Video 2-3, MP4 file.

Developmental delay is a common feature of *STXBP1* encephalopathy (Stamberger et al., 2016; Xian et al., 2022). Given the partial lethality phenotype of Viaat-cHet mice, we sought to examine their developmental milestones. We monitored pinnae detachment, fur development, incision eruption, and eye opening (Heyser, 2004) and found that Viaat-cHet mice developed these milestones similarly to the control mice (Extended Data Fig. 2-1*A*). We also examined the negative geotaxis reflex and surface righting reflex to monitor the development of sensory and motor functions (). The negative geotaxis reflex appeared normal in Viaat-cHet mice (Extended Data Fig. 2-1*B*). The surface righting reflex appeared in all control mouse pups by P7 (Fig. 2*E*), but ∼42% of the Viaat-cHet pups did not flip themselves within 60 s on P7. From P7 to P11, it took longer for Viaat-cHet mice to complete this task than the control mice. Interestingly, those Viaat-cHet pups that later died between P14 and P21 were not obviously different from other survived Viaat-cHet pups (Fig. 2*E*). By P13, Viaat-cHet mice caught up with the control mice (Fig. 2*E*), indicating a developmental delay rather than a loss of this reflex (). During the first three postnatal weeks, the body weight of Viaat-cHet mice including those that later died prematurely was not significantly different from that of their sex- and age-matched control littermates (Extended Data Fig. 2-1*C*) but became consistently less than the control mice after postnatal week 6 (Fig. 2*F*). The body weight of Vglut2-cHet mice was indistinguishable from that of their sex- and age-matched control littermates (Fig. 2*F*). Thus, these results indicated that Viaat-cHet mice are developmentally delayed.

Furthermore, both Viaat-cHet and Vglut2-cHet mice began to develop hindlimb stiffness or clasping ∼3–4 weeks of age when they were picked up by the tail (Fig. 2*G*,*H*; Extended Data Video 2-3), indicating a functional impairment in the cerebellum, basal ganglia, or neocortex (Lalonde and Strazielle, 2011). The phenotype of Viaat-cHet mice was much more severe than that of Vglut2-cHet mice. By the age of 6 months, 78% Viaat-cHet mice developed hindlimb clasping, but only 24% Vglut2-cHet mice did (Fig. 2*I*). To further assess mouse well-being and activities of daily living, we examined the innate nest building behavior (Jirkof, 2014) by providing a Nestlet (i.e., pressed cotton square) to each mouse in the home cage and scoring the degree of shredding and nest quality after 24 h (Fig. 2*J*,*K*). Viaat-cHet mice built worse nests than control mice, similar to *Stxbp1^tm1d/+^* and *Stxbp1^tm1a/+^* mice (Chen et al., 2020), whereas Vglut2-cHet mice were normal (Fig. 2*K*), indicating a change in the health or welfare of Viaat-cHet mice.

Altogether, these results show that *Stxbp1* haploinsufficiency in GABAergic/glycinergic neurons alone is sufficient to recapitulate the reduced survival, developmental delay, hindlimb clasping, and impaired nesting behavior observed in the constitutive *Stxbp1* haploinsufficient mice (Chen et al., 2020).

### *Stxbp1* haploinsufficiency in either GABAergic/glycinergic or glutamatergic neurons increases anxiety-like behaviors, whereas in GABAergic/glycinergic neurons, it causes hyperactivity

**Figure 3.**
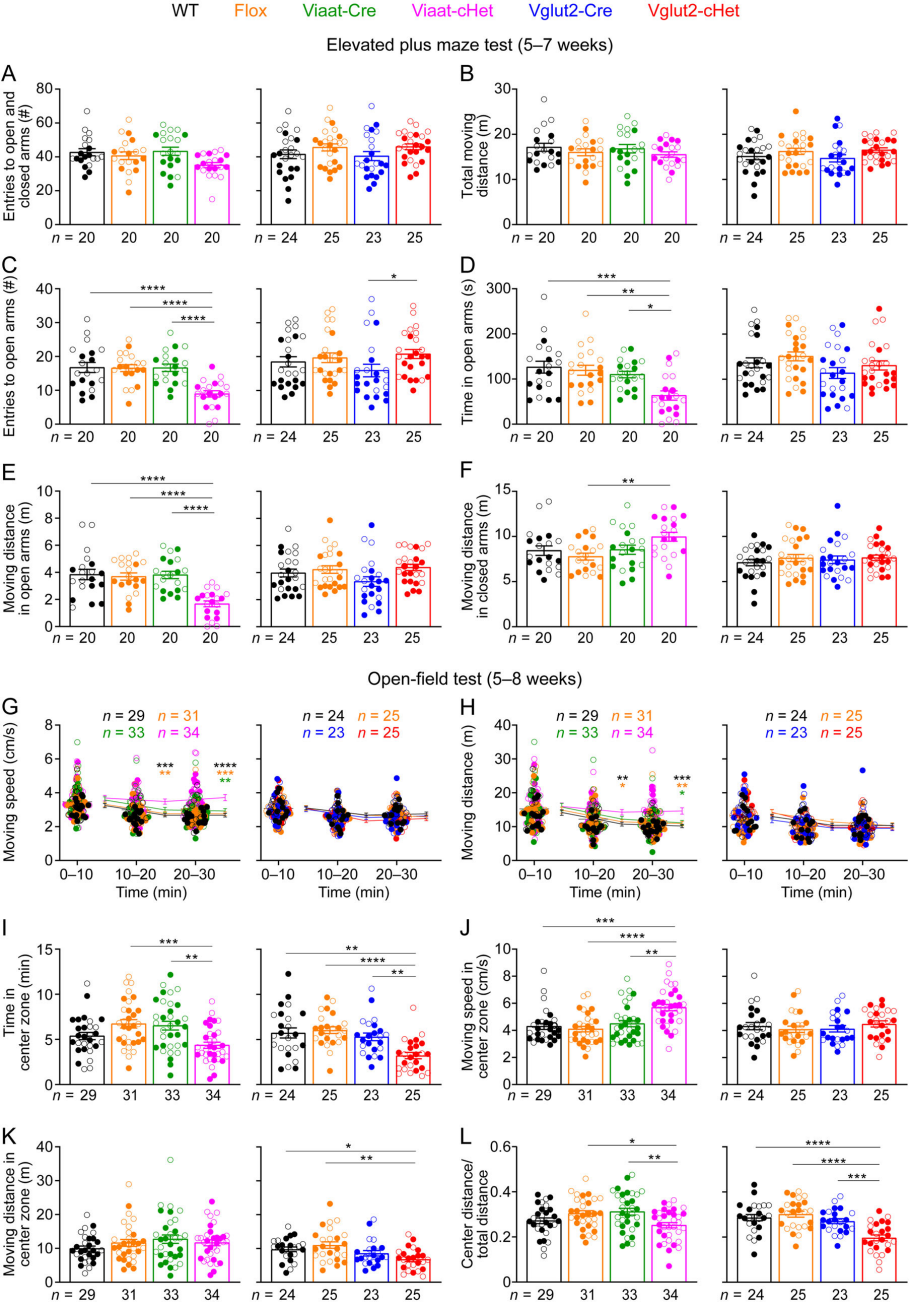
Viaat-cHet mice exhibit heightened anxiety-like behaviors and hyperactivity, whereas Vglut2-cHet mice only display increased anxiety-like behaviors. ***A****–**F***, In the elevated plus maze test, the total entries to the arms (***A***) and travel distance (***B***) of Viaat-cHet and Vglut2-cHet mice were normal. Viaat-cHet mice, but not Vglut2-cHet mice, entered the open arms less frequently (***C***), spent less time (***D***), and traveled shorter distance (***E***) in the open arms than control mice. In the closed arms, the travel distances of Vglut2-cHet mice were similar to those of control mice, and Viaat-cHet mice traveled slightly longer distances than Flox mice (***F***). ***G***, ***H***, In the open-field test, Viaat-cHet mice, but not Vglut2-cHet mice, showed an increase in the moving speed (***G***) and distance (***H***). The statistical significance between Viaat-cHet and WT, Flox, or Viaat-Cre mice is indicated by black, orange, or green asterisks, respectively. ***I–K***, In the center zone of the arena, Viaat-cHet mice spent less time (***I***), moved faster (***J***), and traveled similar distance as the control mice (***K***), whereas Vglut2-cHet mice spent less time (***I***) and traveled shorter distance (***K***). ***L***, Viaat-cHet and Vglut2-cHet mice showed a decrease in the ratio of center moving distances over total moving distance. For different panels, the numbers and ages of tested mice are indicated in the figure. Each filled (male) or open (female) circle represents one mouse. Data are mean ± SEM. *p < 0.05, **p < 0.01, ***p < 0.001, ****p < 0.0001.

Anxiety was reported in a subset of *STXBP1* encephalopathy patients (Marchese et al., 2016; Suri et al., 2017), and several lines of constitutive *Stxbp1* heterozygous knock-out mice including *Stxbp1^tm1d/+^* and *Stxbp1^tm1a/+^* show increased anxiety (Hager et al., 2014; Miyamoto et al., 2017; Kovačević et al., 2018; Chen et al., 2020). Thus, we used the elevated plus maze test to assess anxiety-like behaviors. Overall, Viaat-cHet and Vglut2-cHet mice made similar numbers of entries into the arms and traveled similar distance as their control mice (Fig. 3*A*,*B*). However, Viaat-cHet mice entered the open arms less frequently, spent less time, and traveled shorter distance in the open arms than control mice (Fig. 3*C–E*). In the closed arms, the travel distances are overall similar among different groups (Fig. 3*F*). Thus, Viaat-cHet mice exhibit heightened anxiety-like behaviors, but Vglut2-cHet mice did not show such phenotypes in this test (Fig. 3*C–F*).

We next used the open-field test to examine mouse locomotion, exploration, and anxiety-like behaviors, as *Stxbp1^tm1d/+^* and *Stxbp1^tm1a/+^* mice show hyperactivity and increased anxiety-like behaviors in this test (Chen et al., 2020). Typically, the activities of mice decrease over time in this test, as mice become acclimated to the test arena. The locomotion of Viaat-cHet mice did not decrease over time, and they traveled longer distances and faster than control mice in the later phase of the test (Fig. 3*G*,*H*), showing a hyperactive phenotype. In contrast, Vglut2-cHet mice were similar to control mice (Fig. 3*G*,*H*). Viaat-cHet mice spent less time in the center region of the arena less than control mice (Fig. 3*I*), although they still moved faster (Fig. 3*J*). Therefore, Viaat-cHet mice traveled similar distance in the center region as control mice (Fig. 3*K*) but proportionally explored the center region less than the control mice (Fig. 3*L*), consistent with their heightened anxiety. Interestingly, Vglut2-cHet mice also avoided the arena center region (Fig. 3*I*,*K*,*L*), indicating an increase in anxiety as well.

Taken the results of the elevated plus maze and open-field tests together, both GABAergic/glycinergic and glutamatergic neurons contribute to anxiety-like behaviors. Furthermore, *Stxbp1* haploinsufficiency in GABAergic/glycinergic neurons mediates the hyperactivity phenotype of constitutive *Stxbp1* haploinsufficient mice.

### Impaired motor coordination and normal sensory functions of GABAergic/glycinergic neuron-specific *Stxbp1* haploinsufficient mice

**Figure 4.**
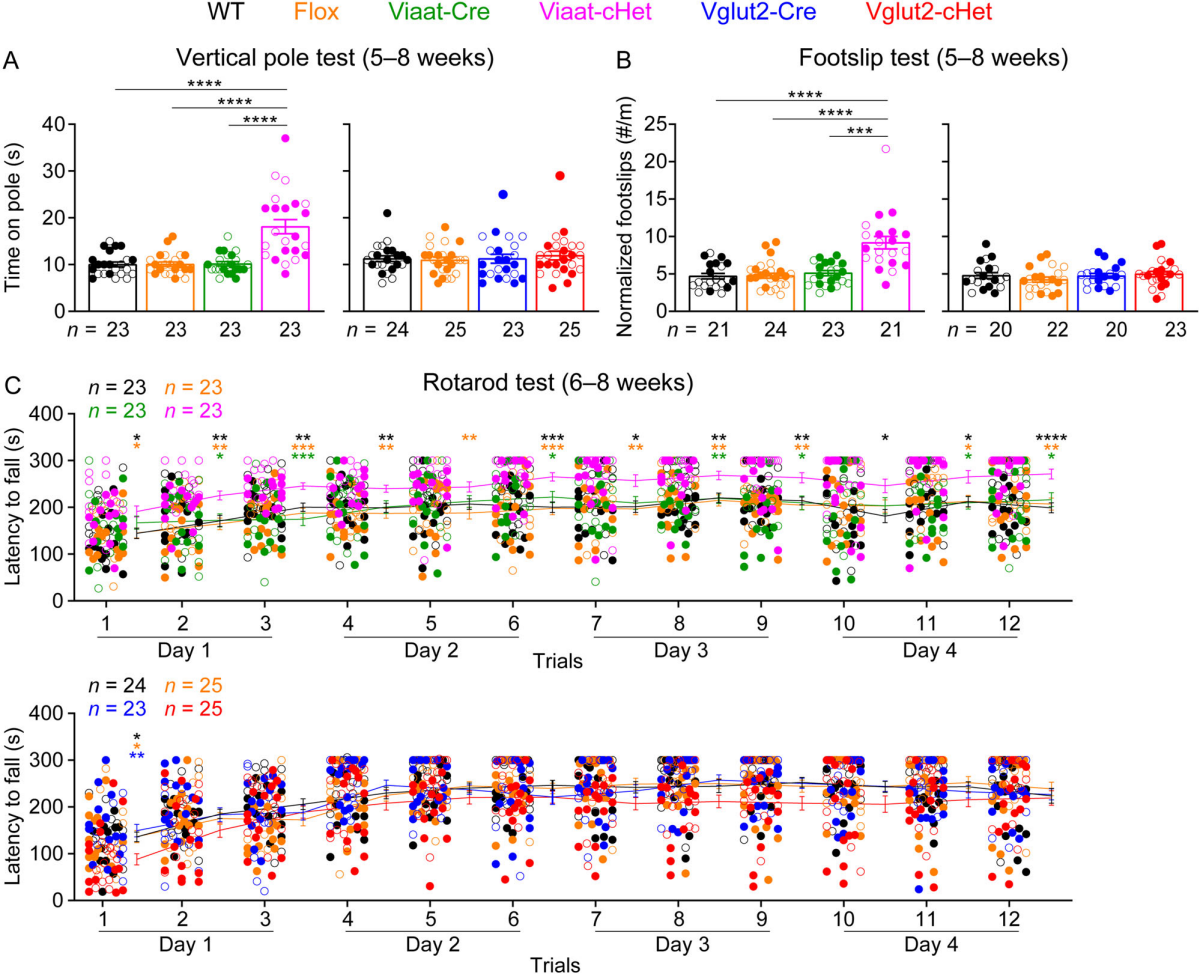
Reduced motor coordination of Viaat-cHet mice. ***A***, ***B***, Viaat-cHet mice, but not Vglut2-cHet mice, took more time to come down from a vertical pole (***A***) and made more foot slips per travel distance when walking on a wire grid (***B***). ***C***, In the rotarod test, Viaat-cHet mice performed better than the control mice, as they were able to stay on the rotating rod for longer time. The statistical significance between Viaat-cHet and WT, Flox, or Viaat-Cre mice is indicated by black, orange, or green asterisks, respectively. Vglut2-cHet mice performed similarly as the control mice except the first trial. The statistical significance between Vglut2-cHet and WT, Flox, or Vglut2-Cre mice is indicated by black, orange, or blue asterisks, respectively. The relationship between rotarod performance and body weight and the results of marble burying test and hole-board test are shown in Extended Data Figure 4-1. For different panels, the numbers and ages of tested mice are indicated in the figure. Each filled (male) or open (female) circle represents one mouse. Data are mean ± SEM. *p < 0.05, **p < 0.01, ***p < 0.001, ****p < 0.0001.

We next evaluated motor functions using vertical pole, foot slip tests, and rotarod, as motor deficits are prevalent in *STXBP1* encephalopathy patients (Stamberger et al., 2016; Xian et al., 2022) and were observed in *Stxbp1^tm1d/+^* and *Stxbp1^tm1a/+^* mice (Chen et al., 2020). The vertical pole test assesses the agility of mice by measuring the amount of time it takes for mice to descend from the top of a vertical pole. Viaat-cHet mice took more time to complete this task than control mice (Fig. 4*A*). When allowed to walk on a wire grid, Viaat-cHet mice had difficulty in placing their paws precisely on the wire to hold themselves and slipped more frequently than control mice (Fig. 4*B*). In contrast, Vglut2-cHet mice performed normally in both vertical pole and foot slip tests (Fig. 4*A*,*B*).

10.1523/JNEUROSCI.1806-23.2024.f4-1 Extended Data Figure 4-1 **Viaat-cHet mice do not show heightened repetitive and stereotyped behaviors.** (**A**) The average latency to fall from the rotating rod across all trials as a function of body weight for Viaat-cHet and their control mice. There are weak but statistically significant negative correlations between the latency and body weight for both male and female mice. (**B**) Similar to (A), but for Vglut2-cHet and their control mice. There are no significant correlations. (**C**) The numbers of buried marbles (i.e., at least 50% of the marble is covered) for Viaat-cHet, Vglut2-cHet, and their control mice. (**D–F**) In the hole-board test, the total numbers of holes that were poked (D) and the total numbers of nose pokes (E) by Viaat-cHet were similar to those by the control mice. The holes were also ranked according to the numbers of repetitive-poke events (i.e., with 2 or more consecutive pokes) in each hole (F). The statistical significance between Viaat-cHet and WT or Viaat-Cre mice is indicated by black or green asterisks, respectively. For different panels, the numbers and ages of tested mice are indicated in the figure. Each filled (male) or open (female) circle represents one mouse. Data are mean ± s.e.m. * *P* < 0.05. Download Extended Data Figure 4-1, TIF file.

We also performed rotarod test for 4 consecutive days to evaluate motor learning and coordination by measuring the latency of mice to fall from a rotating rod. Vglut2-cHet mice performed similarly to control mice except the first trial where they fell off the rotating rod earlier (Fig. 4*C*). In contrast to the expectation, Viaat-cHet mice stayed on the rotating rod longer than the control mice across multiple trials (Fig. 4*C*). This improved performance of Viaat-cHet mice could be due to their hyperactivity and smaller body weight, as rotarod performance was shown to be negatively correlated with body weight (McFadyen et al., 2003; Mao et al., 2015) or an indication of increased repetitive and stereotyped behaviors (Rothwell et al., 2014). To evaluate these two possibilities, we first plotted the rotarod performance as a function of the body weight for each mouse and found only a weak negative correlation for Viaat-cHet and their control mice (Extended Data Fig. 4-1*A*,*B*). To test repetitive and stereotyped behaviors, we performed the marble burying test and hole-board test. The marble burying test evaluates innate digging behavior, and an increase in marble burying could be interpreted as elevated anxiety or repetitive compulsive behaviors. Viaat-cHet mice buried fewer marbles but were not statistically different from the control mice (Extended Data Fig. 4-1*C*). Vglut2-cHet mice buried similar numbers of marbles as the control mice (Extended Data Fig. 4-1*C*). The hole-board test measures the pattern of exploratory nose poke behavior. Viaat-cHet mice explored similar numbers of holes (Extended Data Fig. 4-1*D*), made similar numbers of nose pokes (Extended Data Fig. 4-1*E*), and similar numbers of repetitive-poke events (i.e., two or more consecutive pokes into the same hole; Extended Data Fig. 4-1*F*). Thus, the results of marble burying and hole-board tests are inconsistent with the hypothesis that repetitive and stereotyped behaviors are increased in Viaat-cHet mice. Together, these results show that like constitutive *Stxbp1* haploinsufficient mice, Viaat-cHet mice do not develop ataxia, but their fine motor coordination is impaired. The improved performance of Viaat-cHet mice in the rotarod test is likely the result of their hyperactivity and perhaps smaller body weight.

**Figure 5.**
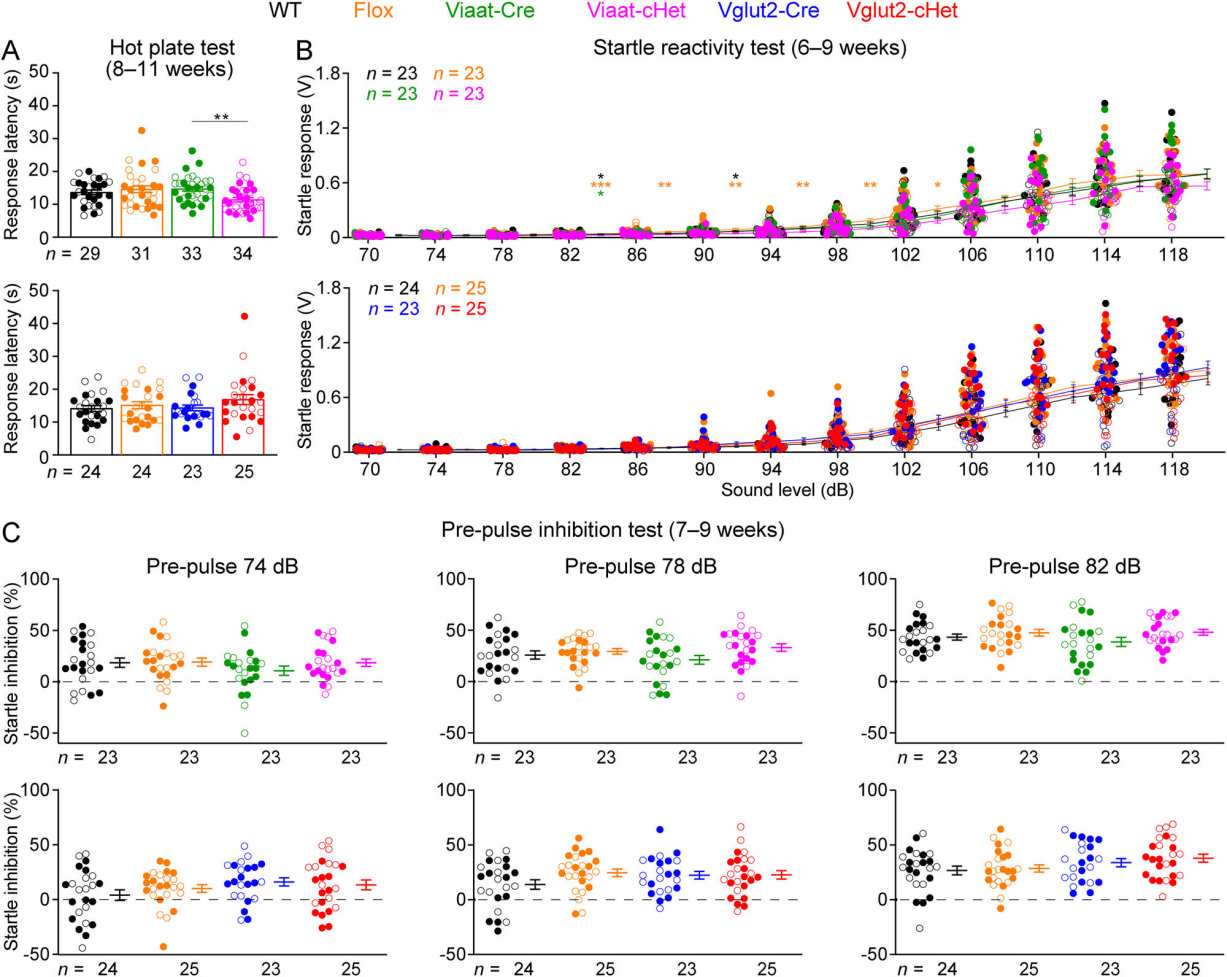
Viaat-cHet and Vglut2-cHet mice have normal sensory functions. ***A***, In the hot plate test, Viaat-cHet mice showed slightly shorter latencies than Viaat-Cre mice in response to the high temperature, and the latencies of Vglut2-cHet were similar to those of control mice. ***B***, Viaat-cHet and Vglut2-cHet mice showed similar acoustic startle responses as the control mice at different sound levels. The statistical significance between Viaat-cHet and WT, Flox, or Viaat-Cre mice is indicated by black, orange, or green asterisks, respectively. ***C***, In the prepulse inhibition test, when a weak sound (i.e., prepulse 74, 78, or 82 dB) preceded a loud sound (120 dB), Viaat-cHet and Vglut2-cHet mice showed a similar reduction in the startle responses to the loud sound as the control mice. For different panels, the numbers and ages of tested mice are indicated in the figure. Each filled (male) or open (female) circle represents one mouse. Data are mean ± SEM. *p < 0.05, **p < 0.01, ***p < 0.001.

We previously showed that *Stxbp1^tm1d/+^* and *Stxbp1^tm1a/+^* mice had normal thermal nociception, acoustic startle responses, and prepulse inhibition (Chen et al., 2020). To determine if GABAergic/glycinergic or glutamatergic neurons-specific *Stxbp1* haploinsufficiency causes additional abnormalities in sensory functions and sensorimotor gating, we examined Viaat-cHet and Vglut2-cHet mice in these assays and found them to be normal (Fig. 5*A–C*).

### Enhanced social aggression in GABAergic/glycinergic neurons-specific *Stxbp1* haploinsufficient mice

**Figure 6.**
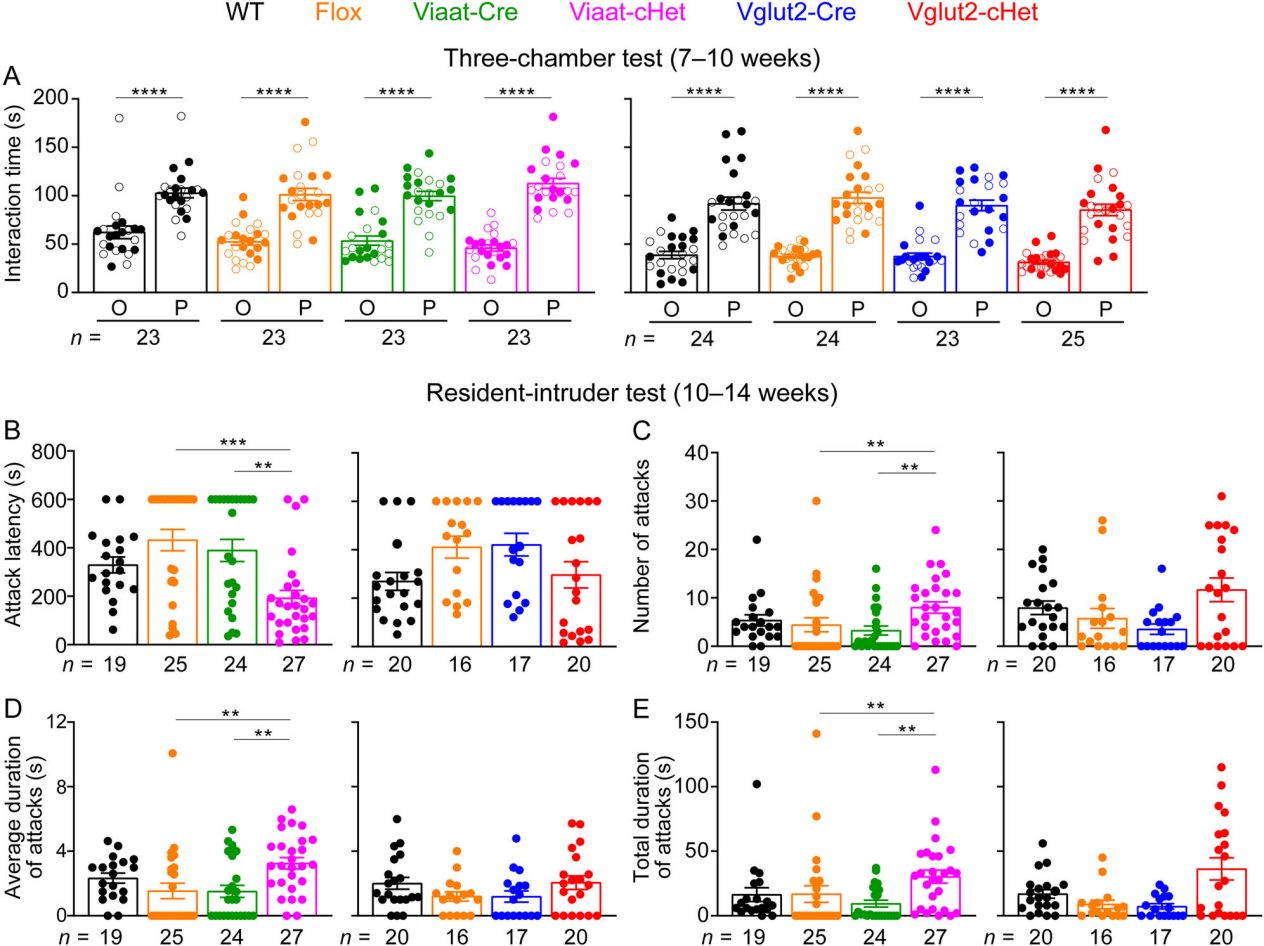
Viaat-cHet mice exhibit normal sociability but increased aggressive behaviors. ***A***, In the three-chamber test, Viaat-cHet, Vglut2-cHet, and the control mice showed a preference for interacting with the partner mouse over the object. ***B****–**E***, In the residentintruder test, male Viaat-cHet mice, but not Vglut2-cHet mice, showed a reduction in the latency to attack the male intruder mice (***B***). The number (***C***), average duration (***D***), and total duration (***E***) of attacks of Viaat-cHet mice were increased as compared with those of the control mice. For different panels, the numbers and ages of tested mice are indicated in the figure. Each filled (male) or open (female) circle represents one mouse. Data are mean ± SEM. **p < 0.01, ***p < 0.001, ****p < 0.0001.

A subset of *STXBP1* encephalopathy patients exhibit autistic features and aggressive behaviors (Stamberger et al., 2016; Abramov et al., 2021). *Stxbp1^tm1d/+^* and *Stxbp1^tm1a/+^* mice show normal social interactions in the three-chamber test, but male resident *Stxbp1^tm1d/+^* and *Stxbp1^tm1a/+^* mice exhibit elevated innate aggression toward male intruder mice in the resident–intruder test (Chen et al., 2020). Thus, we evaluated Viaat-cHet and Vglut2-cHet mice in these two tests. In the three-chamber test, both Viaat-cHet and Vglut2-cHet mice preferred to interact with a sex- and age-matched partner mouse rather than an object, similar to the control mice (Fig. 6*A*), showing their normal sociability. In the resident–intruder test, compared with the control mice, male resident Viaat-cHet mice started the first attack sooner, initiated more attacks, and spent more time attacking the intruders (Fig. 6*B–E*), all of which indicate an elevated innate aggression. In contrast, Vglut2-cHet mice were not statistically different from control mice in any of these parameters, although there might be signs of elevated aggression based on the number of attacks and total duration of attacks (Fig. 6*B–E*). These results indicate that GABAergic**/**glycinergic neurons are critically involved in the elevated innate aggression caused by *Stxbp1* haploinsufficiency.

### GABAergic/glycinergic and glutamatergic neuron-specific *Stxbp1* haploinsufficiency differentially impair conditioned fear memory

**Figure 7.**
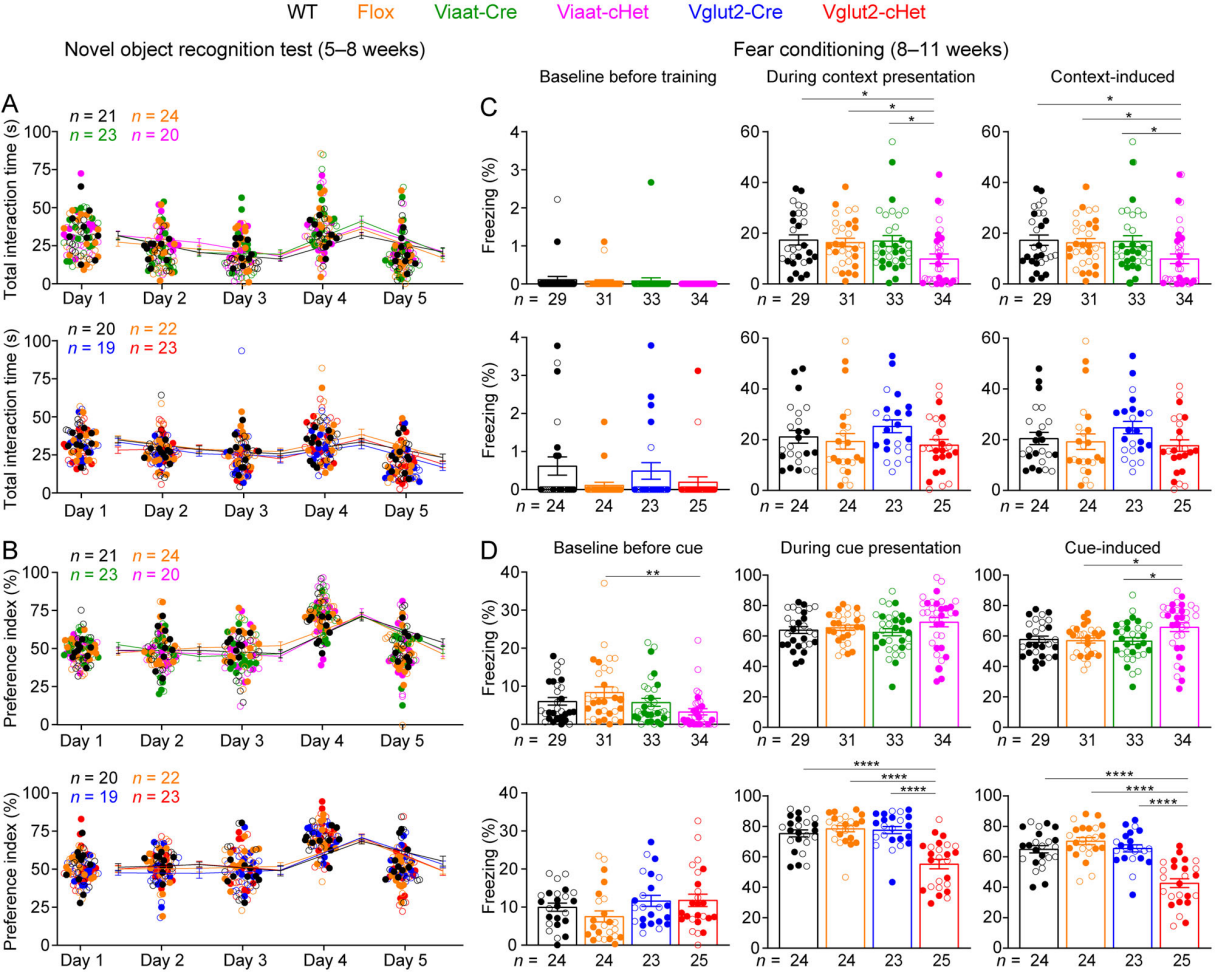
Distinct fear memory deficits of Viaat-cHet and Vglut2-cHet mice. ***A***, ***B***, In the novel object recognition test with 24 h testing intervals, mice were presented with the same two identical objects on days 1, 2, 3, and 5, and the familiar object and a novel object on day 4. The total interaction time with familiar and novel objects of Viaat-cHet or Vglut2-cHet mice was similar to that of their control mice (***A***). The ability of a mouse to recognize the novel object was measured by the preference index (***B***). Viaat-cHet and Vglut2-cHet mice showed similar preference for the novel object as their control mice. ***C***, In fear conditioning, Viaat-cHet mice showed a similar level of freezing behaviors to the control mice before training (left panel) and a reduction of freezing behaviors in the contextual memory test 24 h after training (middle panel). Thus, contextual memory assessed by the context-induced freezing behaviors (right panel) is impaired in Viaat-cHet mice. Vglut2-cHet mice showed no impairments in this memory. ***D***, In the cued memory test 24 h after training, Vglut2-cHet mice showed a similar level of freezing behaviors to the control mice before the cue presentation (left panel) and a reduction of freezing behaviors during cue presentation (middle panel). Thus, cued memory assessed by the cue-induced freezing behaviors (right panel) is impaired in Vglut2-cHet mice. The cue-induced freezing behaviors (right panel) is slightly enhanced in Viaat-cHet mice because Viaat-cHet mice showed a slight reduction of freezing behaviors before the cue presentation (left panel) and similar freezing behaviors to the control mice during the cue presentation (middle panel). The time courses of freezing behaviors are shown in Extended Data Figure 7-1. For different panels, the numbers and ages of tested mice are indicated in the figure. Each filled (male) or open (female) circle represents one mouse. Data are mean ± SEM. *p < 0.05, **p < 0.01, ****p < 0.0001.

One of the core features of *STXBP1* encephalopathy is intellectual disability (Stamberger et al., 2016; Abramov et al., 2021; Xian et al., 2022), which is recapitulated by the severe cognitive deficits in *Stxbp1^tm1d/+^* and *Stxbp1^tm1a/+^* mice (Chen et al., 2020). To assess the cognitive functions of Viaat-cHet and Vglut2-cHet mice, we first performed the novel object recognition test, in which WT mice prefer to explore a novel object over a familiar object, whereas *Stxbp1^tm1d/+^* and *Stxbp1^tm1a/+^* mice fail to recognize the novel object (Chen et al., 2020). Neither Viaat-cHet nor Vglut2-cHet mice showed a deficit in this test, as they had similar interaction time and preference index as the control mice (Fig. 7*A*,*B*). This result was unexpected because novel object recognition is thought to depend on the hippocampus and cortex (Antunes and Biala, 2012; Cohen and Stackman, 2015), and *Stxbp1* haploinsufficiency impairs GABAergic and glutamatergic synaptic transmission (Toonen et al., 2006; Patzke et al., 2015; Orock et al., 2018; Miyamoto et al., 2019; Chen et al., 2020; Dos Santos et al., 2023), which is expected to alter cortical functions. Thus, the intact recognition memories in Viaat-cHet and Vglut2-cHet mice indicate that *Stxbp1* haploinsufficiency in GABAergic/glycinergic or glutamatergic neurons alone is not sufficient to impair novel object recognition or other neuronal types are more important for this cognitive function.

10.1523/JNEUROSCI.1806-23.2024.f7-1 Extended Data Figure 7-1 **The time courses of freezing behaviors in fear conditioning.** (**A**) The freezing behaviors of Viaat-cHet and control mice as a function of time during training (upper panel), contextual memory test (middle panel), and cued memory test (lower panel) conducted 24 hours after training. During training, Viaat-cHet mice did not freeze as much as the control mice after the first and second sound presentations but froze similarly in response to the second sound presentation. The statistical significance between Viaat-cHet and WT, Flox, or Viaat-Cre mice is indicated by black, orange, or green asterisks, respectively. (**B**) Similar to (A), but for Vglut2-cHet and control mice. During training, Vglut2-cHet mice did not freeze as much as the control mice in response to the second sound presentation. The statistical significance between Vglut2-cHet and WT, Flox, or Vglut2-Cre mice is indicated by black, orange, or blue asterisks, respectively. (**C,D**) Similar to (A), but for *Stxbp1^tm1d/+^*, *Stxbp1^tm1a/+^*, and control mice. During training, *Stxbp1^tm1d/+^* and *Stxbp1^tm1a/+^* mice did not freeze as much as the control mice after the first and second sound presentations and in response to the second sound presentation. For different panels, the numbers and ages of tested mice are indicated in the figure. Data are mean ± s.e.m. * *P* < 0.05, ** *P* < 0.01, *** *P* < 0. 001, **** *P* < 0. 0001. Download Extended Data Figure 7-1, TIF file.

To further examine cognitive functions, we evaluated Viaat-cHet and Vglut2-cHet mice in the Pavlovian fear conditioning paradigm, in which *Stxbp1^tm1d/+^* and *Stxbp1^tm1a/+^* mice display a strong reduction in both context-induced (i.e., the environment) and cue-induced (i.e., the sound) freezing behaviors 24 h after conditioning (Chen et al., 2020). Interestingly, Viaat-cHet and Vglut2-cHet mice showed a selective deficit in hippocampus-dependent contextual fear memory and hippocampus-independent cued fear memory, respectively (Fig. 7*C*,*D*). Vglut2-cHet mice were normal in contextual memory (Fig. 7*C*), whereas Viaat-cHet mice even had slightly better cued memory than control mice as they showed a reduction in freezing responses before the onset of cue and similar responses during the cue presentation as the control mice (Fig. 7*D*). The reduced freezing responses in Vglut2-cHet and Viaat-cHet mice were not due to sensory dysfunctions as their acoustic startle responses and nociception were intact (Fig. 5). Inspecting the freezing behaviors during the training phase revealed that Viaat-cHet mice had reduced freezing responses after the first footshock and normal responses to the second sound (Extended Data Fig. 7-1*A*), whereas Vglut2-cHet mice had normal responses after the first footshock and reduced responses to the second sound (Extended Data Fig. 7-1*B*). Thus, Viaat-cHet mice can form normal association between the cue and footshocks, but not between the context and footshocks. In contrast, Vglut2-cHet mice were normal in associating the context and footshocks, but not between the cue and footshocks. We also analyzed the freezing behaviors during the training phase of *Stxbp1^tm1d/+^* and *Stxbp1^tm1a/+^* mice from Chen et al. (2020) and found that neither mutant exhibited the normal ability to form either association (Extended Data Fig. 7-1*C*,*D*). Thus, Viaat-cHet and Vglut2-cHet mice each recapitulate one aspect of the learning deficits in the constitutive *Stxbp1* haploinsufficient mice. This segregation of two forms of associative learning and memory in Viaat-cHet and Vglut2-cHet mice highlights the importance of both GABAergic/glycinergic and glutamatergic neurons in the cognitive deficits of *STXBP1* encephalopathy.

### Distinct epileptic seizures in GABAergic/glycinergic and glutamatergic neuron-specific *Stxbp1* haploinsufficient mice

**Figure 8.**
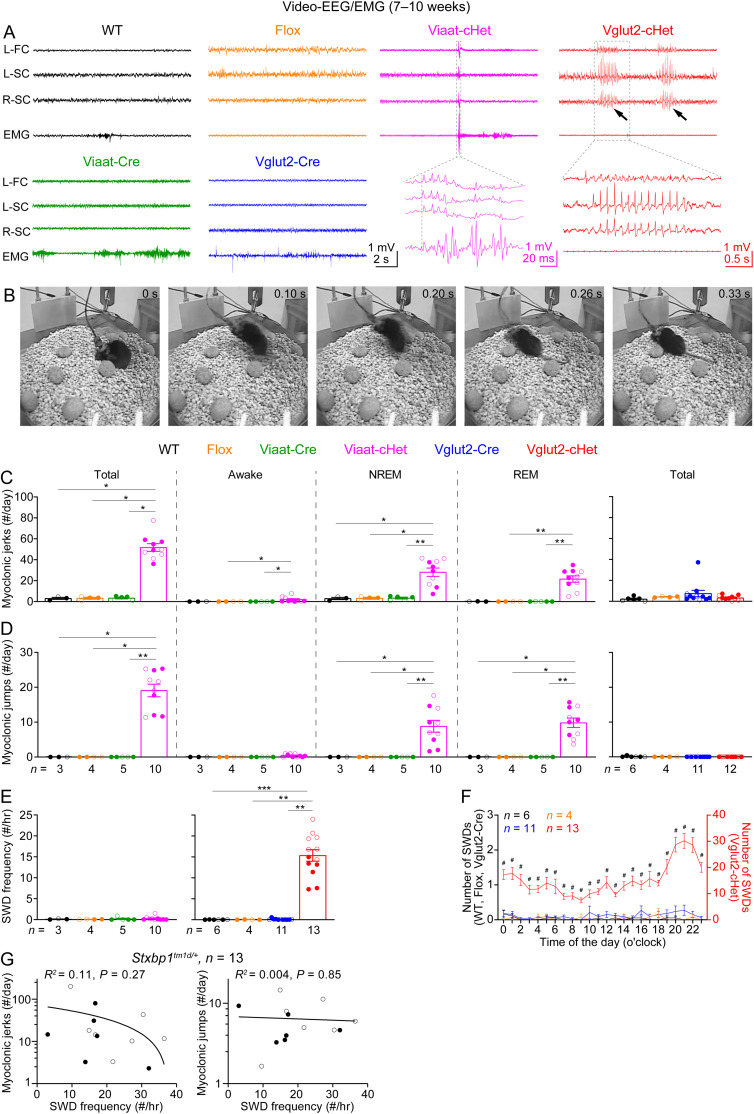
Viaat-cHet and Vglut2-cHet mice exhibit different forms of epileptic seizures. ***A***, Representative EEG traces from the left frontal cortex (L-FC), left somatosensory cortex (L-SC), right somatosensory cortex (*R*-SC), and EMG traces from the neck muscle. A myoclonic jerk from a Viaat-cHet mouse was indicated by the dashed box and was expanded to show that the EEG discharges occurred prior to the EMG discharges. Note, the vertical line marks the onset of EEG discharges. The mouse was in REM sleep before the jerk (Extended Data Video 8-1). Two SWDs from a Vglut2-cHet mouse were indicated by the arrows and one of them was expanded below (Extended Data Video 8-2). ***B***, Video frames showing a myoclonic jump of a Viaat-cHet mouse (Extended Data Video 8-3). The mouse was in REM sleep before the jump. ***C***, Summary data showing the total frequencies of myoclonic jerks and the frequencies in different behavioral states. The frequency of jerks was drastically increased in Viaat-cHet mice, particularly during NREM and REM sleep. ***D***, Similar to ***C***, but for myoclonic jumps. The frequency of jumps was drastically increased in Viaat-cHet mice, particularly during NREM and REM sleep. ***E***, Summary data showing that the SWD frequencies of Vglut2-cHet mice were drastically increased as compared with those of the control mice. ***F***, The numbers of SWDs per hour in control (left *y*-axis) and Vglut2-cHet (right *y*-axis) mice are plotted as a function of time of day and averaged over 3 d. ***G***, The relationships between the SWD frequency and the total frequency of myoclonic jerks (left panel) or jumps (right panel) in the *Stxbp1^tm1d/+^* mice from Chen et al. (2020) were fitted with a linear regression (*Y* *=* *aX + b*; *X*, SWD frequency; *Y*, jerk or jump frequency; *a*, *b*, constants). The SWD frequency is not correlated with the total frequency of myoclonic jerks or jumps. The results of *Stxbp1* haploinsufficiency in different classes of inhibitory neurons are shown in Extended Data Figure 8-4. For different panels, the numbers and ages of recorded mice are indicated in the figure. Each filled (male) or open (female) circle represents one mouse. Data are mean ± SEM. *P < 0.05, **p < 0.01, ***p < 0.001.

Epilepsy is a hallmark feature of *STXBP1* encephalopathy, and patients present diverse seizure types including epileptic spasm, focal, tonic, clonic, myoclonic, and absence seizures (Stamberger et al., 2016; Suri et al., 2017). Constitutive *Stxbp1* heterozygous knock-out mice including *Stxbp1^tm1d/+^* mice have frequent SWDs, the hallmark of absence seizures, as well as myoclonic seizures that manifest as involuntary muscle jerks associated with EEG discharges or a more severe form—sudden jumps (Kovačević et al., 2018; Miyamoto et al., 2019; Chen et al., 2020). Although a subset of observed myoclonic jerks are probably physiological because WT mice also show a small number of these events, *Stxbp1^tm1d/+^* mice have many more, particularly during sleep (Chen et al., 2020). We performed chronic video-EEG/EMG recordings in freely moving Viaat-cHet, Vglut2-cHet, and control mice (Fig. 8*A*,*B*). All Viaat-cHet mice showed many myoclonic jerks and jumps, particularly during REM and NREM sleeps (Fig. 8*A–D*; Extended Data Video 8-1; Extended Data Video 8-2), but interestingly did not have more SWDs than the control mice (Fig. 8*E*). In contrast, all Vglut2-cHet mice exhibited numerous SWDs (Fig. 8*A*,*E*; Extended Data Video 8-3), but their myoclonic jerks and jumps were indistinguishable from those of control mice (Fig. 8*C*,*D*). The SWDs in Vglut2-cHet mice occurred throughout the day and night and at higher frequencies during the night (Fig. 8*F*), which is consistent with what we observed in *Stxbp1^tm1d/+^* mice and the notion that absence seizures typically occur during the awake state (Panayiotopoulos, 2008). The distinct seizure phenotypes between Viaat-cHet and Vglut2-cHet mice suggest that SWDs and myoclonic seizures likely involve different neural circuits and are independent from each other. Consistent with this notion, the frequencies of SWDs and myoclonic seizures in *Stxbp1^tm1d/+^* mice from Chen et al. (2020) do not correlate with each other across mice (Fig. 8G). Thus, the segregation of two types of seizures in Viaat-cHet and Vglut2-cHet mice highlights the important, but different, roles of GABAergic/glycinergic and glutamatergic neurons in the epileptogenesis for *STXBP1* encephalopathy.

10.1523/JNEUROSCI.1806-23.2024.f8-4 Extended Data Figure 8-4 ***Stxbp1* haploinsufficiency in subtypes of GABAergic neurons.** (**A**) Summary data showing the total frequencies of myoclonic jerks (left panel) and jumps (right panel) from Pv-cHet (*Stxbp1^f/+^;Pv^Cre/+^*) and control Pv-Cre (*Pv^Cre/+^*) mice. (**B,C**) Similar to (A), but for Sst-cHet (*Stxbp1^f/+^;Sst^Cre/+^*) and control Sst-Cre (*Sst^Cre/+^*) mice (B) and Htr3a-cHet (*Stxbp1^f/+^;Htr3a-Cre^Tg/+^*) and control Htr3a-Cre (*Htr3a-Cre^ Tg/+^*) mice (C). (**D**) Representative fluorescence images of a sagittal brain section from *Dlx5/6-Cre^Tg/+^;Rosa26^tdTomato/+^* mice (*n* = 2, P92 and P101) labeled by ISH probes against *tdTomato* and *Gad1* and a nuclear marker DAPI. *tdTomato* expression is largely restricted to forebrain *Gad1*-positive cells. (**E**) Summary data showing the total frequencies of myoclonic jerks and the frequencies in different behavioral states (left panel) and total frequencies of myoclonic jumps (right panel) from Dlx5/6-cHet (*Stxbp1^f/+^;Dlx5/6-Cre^Tg/+^*) and control Dlx5/6-Cre (*Dlx5/6-Cre^Tg/+^*) mice. For different panels, the numbers and ages of recorded mice are indicated in the figure. Each filled (male) or open (female) circle represents one mouse. Data are mean ± s.e.m. * *P* < 0.05. Download Extended Data Figure 8-4, TIF file.

10.1523/JNEUROSCI.1806-23.2024.video.8-1 Extended Data Video 8-1 **Myoclonic jerks in Viaat-cHet mice.** A representative video showing a myoclonic jerk of a Viaat-cHet mouse. The top 3 traces are EEG signals from the left frontal cortex, right somatosensory cortex, and left somatosensory cortex. The bottom trace is the EMG signal from the neck muscle. The vertical line indicates the time of the current video frame. The mouse was in REM sleep before the jerk. Note that the EEG signal from the left somatosensory cortex (the third channel) is inverted. Download Extended Data Video 8-1, MP4 file.

10.1523/JNEUROSCI.1806-23.2024.video.8-2 Extended Data Video 8-2 **SWDs in Vglut2-cHet mice.** A representative video showing several SWDs in a Vglut2-cHet mouse. The top 3 traces are EEG signals from the left frontal cortex, right somatosensory cortex, and left somatosensory cortex. The bottom trace is the EMG signal from the neck muscle. The vertical line indicates the time of the current video frame. Note that the EEG signal from the left somatosensory cortex (the third channel) is inverted. Download Extended Data Video 8-2, MP4 file.

10.1523/JNEUROSCI.1806-23.2024.video.8-3 Extended Data Video 8-3 **Myoclonic jumps in Viaat-cHet mice.** A representative video showing a myoclonic jump of a Viaat-cHet mouse. The top 3 traces are EEG signals from the left frontal cortex, right somatosensory cortex, and left somatosensory cortex. The bottom trace is the EMG signal from the neck muscle. The vertical line indicates the time of the current video frame. The mouse was in REM sleep before the jump. Note that the EEG signal from the left somatosensory cortex (the third channel) is inverted. Download Extended Data Video 8-3, MP4 file.

The increase of SWDs in Vglut2-cHet mice is consistent with the previous result that heterozygous deletion of *Stxbp1* in dorsal telencephalic glutamatergic neurons caused frequent SWDs (Miyamoto et al., 2019), indicating that *Stxbp1* haploinsufficiency in cortical glutamatergic neurons is sufficient to cause SWDs. The specific types of inhibitory neurons mediating myoclonic jerks and jumps are unknown, but these myoclonic seizures can involve both forebrain and hindbrain (Lalonde and Strazielle, 2012). To further understand the cellular origins of myoclonic seizures, we first generated *Stxbp1* haploinsufficiency selectively in parvalbumin (Pv), somatostatin (Sst), or serotonin receptor 3A (Htr3a)-expressing neurons because at least in the cortex, inhibitory neurons can be classified into one of these three nonoverlapping types, each of which accounts for ∼30–40% of GABAergic neurons (Rudy et al., 2011). We used three well-established Cre lines, *Pv-ires-Cre* (Hippenmeyer et al., 2005), *Sst-ires-Cre* (Taniguchi et al., 2011), and *Htr3a-Cre* (Gerfen et al., 2013; Miyoshi et al., 2015), to target these neuronal types. Interestingly, the survival, body weight, and hindlimb clasping of Pv-cHet (*Stxbp1^f/+^;Pv^Cre/+^*), Sst-cHet (*Stxbp1^f/+^;Sst^Cre/+^*), and Htr3a-cHet (*Stxbp1^f/+^;Htr3a-Cre^Tg/+^*) mice seem normal (data not shown). None of them showed more myoclonic jerks or jumps than their control mice (Extended Data Fig. 8-4*A–C*), indicating that myoclonic seizures are not caused by *Stxbp1* haploinsufficiency in one particular type of GABAergic/glycinergic neurons. We then used a *Dlx5/6-Cre* line (Monory et al., 2006) to determine if *Stxbp1* haploinsufficiency in forebrain GABAergic neurons is sufficient to cause myoclonic seizures. We confirmed the forebrain expression pattern of *Dlx5/6-Cre* by DFISH in *Dlx5/6-Cre^Tg/+^;Rosa26^tdTomato/+^* mice (Extended Data Fig. 8-4*D*). Video-EEG/EMG recordings showed that Dlx5/6-cHet mice (*Stxbp1^f/+^;Dlx5/6-Cre^Tg/+^*) had frequent myoclonic seizures but manifested with only jerks and no jumps. Interestingly, most jerks occurred in NREM sleep (Extended Data Fig. 8-4*E*). This result demonstrates the important role of forebrain GABAergic neurons in the epileptogenesis of *STXBP1* encephalopathy but also suggests that additional GABAergic/glycinergic neurons in the hindbrain or spinal cord may be involved in the generation of myoclonic jumps.

## Discussion

**Figure 9.**
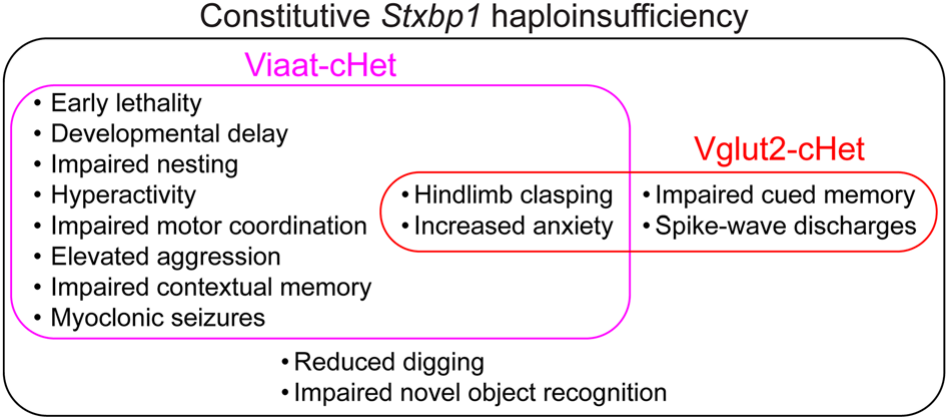
Phenotypic comparison of constitutive *Stxbp1* haploinsufficient mice, Viaat-cHet mice, and Vglut2-cHet mice. Square Venn diagram showing the phenotypes of constitutive *Stxbp1* haploinsufficient mice, Viaat-cHet mice, and Vglut2-cHet mice. Except the reduced digging behavior and impaired novel object recognition, Viaat-cHet and Vglut2 mice together recapitulate all other phenotypes of constitutive haploinsufficient mice. Viaat-cHet and Vglut2-cHet mice each recapitulate distinct subsets of the phenotypes of constitutive haploinsufficient mice, and only hindlimb clasping and increased anxiety are shared between them. Viaat-cHet mice exhibit broader and more severe phenotypes than Vglut2-cHet mice. The phenotypic comparison of different mouse models is shown in Extended Data Table 9-1.

*Stxbp1* haploinsufficiency in GABAergic/glycinergic neurons results in a spectrum of phenotypes including early lethality, developmental delay, impaired nest building, hyperactivity, motor dysfunction, aggression, impaired contextual fear memory, myoclonic seizures, hindlimb clasping, and anxiety-like behaviors. In contrast, haploinsufficiency in glutamatergic neurons leads to impaired cued fear memory, SWDs, hindlimb clasping, and anxiety-like behaviors (Fig. 9; Extended Data Table 9-1). Thus, dysfunctions in GABAergic/glycinergic and glutamatergic neurons mediate distinct neurological impairments of *STXBP1* encephalopathy, but GABAergic/glycinergic neurons are likely the most critical cell type because they mediate majority of the phenotypes. Our results are based on genetic manipulations in vivo and support the hypothesis that inhibitory dysfunction is a primary mechanism of *STXBP1* encephalopathy (Chen et al., 2020). This conclusion is in contrast with prior studies that implied a major role of impaired excitatory synaptic transmission in the disease pathogenesis (Patzke et al., 2015; Miyamoto et al., 2017, 2019; Orock et al., 2018; Dos Santos et al., 2023), which was based on both reduced excitatory synaptic transmission in constitutive heterozygous knock-out neurons and limited neurological phenotypes of the previous models deleting one copy of *Stxbp1* in inhibitory neurons (Extended Data Table 9-1). The first model used a *vesicular inhibitory amino acid transporter (Viaat)-Cre* line to delete *Stxbp1* exon 3, and the mutant mice showed twitches and jumps, but normal survival, locomotion, fear memory, and innate aggression (Miyamoto et al., 2017, 2019). The second model used a *glutamic acid decarboxylase 2 (Gad2)-ires-Cre* line to delete *Stxbp1* exon 2, and the mutant mice showed partial early lethality and epileptiform activities, but other neurological functions were not studied (Kovačević et al., 2018). Apart from different experimental conditions or assays that may have contributed to the differences among studies, another possible difference among these models is the efficiency and specificity of *Stxbp1* deletion resulted from different *Cre* lines and different *Stxbp1* flox alleles, but the conditional deletion of *Stxbp1* was not quantified in previous studies.

10.1523/JNEUROSCI.1806-23.2024.t9-1 Extended data Table 9-1 **Phenotypic comparison of human patients and different mouse models.** The phenotyping tests in different mouse models (the second column) are grouped based on the clinical features of *STXBP1* encephalopathy (the first column). The results of the phenotyping tests from different mouse models are compared in the table. Download Extended data Table 9-1, DOCX file.

Of the phenotypes observed in constitutive *Stxbp1* haploinsufficient mice, deficits in marble burying and novel object recognition are the only two that were not recapitulated by either Viaat-cHet or Vglut2-cHet mice (Fig. 9; Extended Data Table 9-1). One possibility is that other neuronal types such as neuromodulatory systems are important for these phenotypes. Another possibility is the presence of compensatory effects in Viaat-cHet or Vglut2-cHet mice that were not in the constitutive *Stxbp1* haploinsufficient mice. For example, the reduction of Stxbp1 protein levels in the GABAergic/glycinergic neurons of Viaat-cHet mice may not be as great as that in *Stxbp1^tm1d/+^* mice, even though conditional recombination converts *tm1c* to *tm1d* allele. Nevertheless, the latter possibility would have strengthened the critical role of inhibitory neurons in *STXBP1* encephalopathy.

Excitatory and inhibitory neurons are extensively interconnected throughout the brain, and their synaptic interactions control the spatiotemporal patterns of neuronal activity and brain functions (Haider and Mccormick, 2009; Isaacson and Scanziani, 2011). Thus, one would expect that *Stxbp1* dysfunction, which affects synaptic transmission in both cell types, in either neuronal population should lead to largely overlapping neurological phenotypes. On the contrary, our cell type-specific deletion studies reveal distinct roles of these two neuronal types in the phenotypic spectrum of *STXBP1* encephalopathy, and the only shared phenotypes between Viaat-cHet and Vglut2-cHet mice are hindlimb clasping and increased anxiety-like behaviors (Fig. 9; Extended Data Table 9-1). Intriguingly, GABAergic/glycinergic and glutamatergic neurons each independently subserves one of the two forms of associative memories in Pavlovian fear conditioning and one of the two seizure types. The selective presence of myoclonic seizures in Viaat-cHet mice is consistent with the clinical and experimental data that impaired GABAergic synaptic transmission in the cortex, hippocampus, cerebellum, or basal ganglia can cause myoclonus (Lalonde and Strazielle, 2012), whereas the SWDs in Vglut2-cHet mice may be due to the impaired cortico-striatal glutamatergic neurotransmission (Miyamoto et al., 2019). In contrast, contextual and cued fear memories are controlled by both excitatory and inhibitory neurons in a distributed network including, but not limiting to, the hippocampus, amygdala, and medial prefrontal cortex (Tovote et al., 2015; Palchaudhuri et al., 2024; Singh and Topolnik, 2023). Thus, the phenotypic segregation between excitatory and inhibitory neurons suggests that different neurological functions exhibit different susceptibilities to the presynaptic dysfunctions of GABAergic/glycinergic and glutamatergic neurons. Consistent with this hypothesis, in the mouse models of another synaptic vesicle cycle disorder caused by mutations in *DNM1* (dynamin 1), several neurological phenotypes were also segregated in different neuronal types (Asinof et al., 2015).

The use of *Viaat-ires-Cre* and *Vglut2-ires-Cre* allowed us to identify the distinct roles of GABAergic/glycinergic and glutamatergic neurons in the pathogenesis of *STXBP1* encephalopathy but also has several limitations. First, *Viaat-ires-Cre* targets a molecularly and functionally diverse population of neurons and did not allow us to distinguish the contribution of each subtype of inhibitory neurons to different phenotypes. For example, both GABAergic and glycinergic neurons are targeted by *Viaat-ires-Cre*. Glycinergic neurons are present in the brainstem, spinal cord, and retina and can affect motor, auditory, and visual functions. Thus, glycinergic dysfunction could also contribute to the neurological phenotypes of Viaat-cHet mice. Since *Dlx5/6-Cre* does not target the hindbrain inhibitory neurons, and Dlx5/6-cHet mice only show myoclonic jerks but not jumps, it is possible that the hindbrain inhibitory neurons including the brainstem and spinal cord glycinergic neurons may critically contribute to myoclonic jumps. Furthermore, *Stxbp1* haploinsufficiency in Pv, Sst, or Htr3a neurons alone did not cause epilepsy, which suggests that the myoclonic seizures in Viaat-cHet mice result from the collective action of either more than one specific type of GABAergic/glycinergic neurons or simply >30–40% of GABAergic/glycinergic neurons regardless the type. Future studies should determine if they exhibit any other neurodevelopmental phenotypes. It would also be worth determining to what extent the neurodevelopmental deficits of constitutive *Stxbp1* haploinsufficient mice can be rescued by restoring *Stxbp1* expression solely in GABAergic/glycinergic neurons. The outcomes of these future studies can help further define the critical role of GABAergic/glycinergic neurons in disease pathogenesis and therapeutic development. Second, the separation of glutamatergic or GABAergic neurons from each other and other neuronal types based on the neurotransmitters is not absolute, as some neuronal populations use more than a single chemical transmitter (El Mestikawy et al., 2011; Vaaga et al., 2014). For example, forebrain cholinergic neurons express Viaat and release both acetylcholine and GABA (Saunders et al., 2015; ). Vglut2 is expressed in some dopaminergic neurons of the substantia nigra and ventral tegmental area, and these neurons release both glutamate and dopamine (Dal Bo et al., 2008; Mendez et al., 2008; El Mestikawy et al., 2011; Steinkellner et al., 2018; Cai and Tong, 2022). Thus, cholinergic and dopaminergic transmission may also contribute to the phenotypes of Viaat-cHet and Vglut2-cHe mice, respectively. Future studies of conditional *Stxbp1* haploinsufficiency in cholinergic or dopaminergic neurons specifically can help address this question.

The reduction of glutamatergic and GABAergic/glycinergic synaptic transmission caused by *STXBP1* or *Stxbp1* heterozygous mutations are rather modest (Toonen et al., 2006; Patzke et al., 2015; Orock et al., 2018; Miyamoto et al., 2019; Chen et al., 2020; Dos Santos et al., 2023), yet the neurological impairments in humans and mice are severe (Stamberger et al., 2016; Chen et al., 2020; Xian et al., 2022), highlighting the profound impacts of presynaptic dysfunctions on neuronal functions. *STXBP1* encephalopathy shares the core clinical features with other synaptic vesicle cycle disorders, including intellectual disability, epilepsy, and motor dysfunctions. Thus, understanding the cellular and circuit origins of this disorder not only provides mechanistic insights into the growing list of neurodevelopmental disorders caused by presynaptic dysfunctions but also guides the development of therapeutic interventions. The distinct roles of GABAergic/glycinergic and glutamatergic neurotransmitter systems in the disease pathogenesis present both challenges and opportunities. Both neuronal types should be the targets, but their wide distribution throughout the brain makes it challenging for gene-based therapies such as adeno-associated virus–mediated gene therapy to achieve a high degree of coverage for both populations. Nevertheless, the inhibitory neurons should be the primary target given their more critical roles. On the other hand, the clinical symptoms vary considerably among patients, and some patients present only a subset of disease phenotypes (Xian et al., 2022). Thus, regulating one of these two neurotransmitter systems by small molecules such as transmitter receptor modulators may allow more precise treatment of the symptoms.
