# Supplementary material for: GABAergic/Glycinergic and Glutamatergic Neurons Mediate Distinct Neurodevelopmental Phenotypes of STXBP1 Encephalopathy
Source: J Neurosci. 2024 Feb 15;44(14):e1806232024. doi: 10.1523/JNEUROSCI.1806-23.2024 (PMC10993039; doi:10.1523/JNEUROSCI.1806-23.2024)
Supplement: Extended Data List 1-1 — Double fluorescence in situ hybridization probe sequences The sequences of the Stxbp1, Gad1, Vglut1, Vglut2, and tdTomato probes are provided. Download Extended Data List 1-1, DOCX file. [file jneuro-44-e1806232024-s001.docx]

*Stxbp1* probe sense sequence (Allen Brain Atlas, NCBI Accession NM_009295.1):

GCGTCCTTCAGCACCACTGCTGTGAGTGCCCGCTATGGACATTGGCACAAGAATAAGGCCCCCGGGGAGTACCGCAGCGGTCCCCGCCTCATTATTTTCATCCTTGGGGGTGTGAGCCTGAATGAGATGCGCTGTGCTTACGAAGTGACCCAGGCCAACGGCAAGTGGGAAGTGCTGATAGGTTCTACTCACATTCTCACTCCCACCAAATTCCTCATGGACCTGAGACACCCCGACTTCAGGGAGTCCTCTAGGGTATCTTTTGAGGATCAGGCTCCAACAATGGAGTGAGAGCCAAAGAGACAAAGATCCACGCACATTCTCACCCCACAGAAACTGCTGGACACGCTGAAGAAGCTGAATAAAACAGATGAAGAAATAAGCAGTTAAAAAATAAGCTGCCCCCCAAAACCCCGGCTCCCTTCCCAAAATGCTCTGCAGCTCCCCCGTGCGCCACCTCGGTTACTCTGCTGCCTCCCCAGCCCTGCACGCCCTGGCCACCCCGTTGCCGTGCTGAGTTCTTCTCCTGTGCGATGACACCCCATCTTGTCCTCTGAAAAGCAAGAGAGTAATGTGTTGTTTTTTAAAAATGAGCATCTTCTGTATGTATCCCACAGTAAGTTCACATGCAAGCTCCACACTGCAGAAGCGTCAGAACTCCGGACCGAGTGAATTCTCCCTTATTTATGACCCCGTGACCTGTATATAGCCCTGTCCCGCGTGTGCACATTGCTTGAATATGGAAAGGTAGATGTGTGGGTGTCTCTCCAAGCTTGGTTGGATTCATTTCTGTCCTTGTTGGTGTTTGTTCCCCGGATAGGACATGCT

*Gad1* probe sequence (Eurexpress probe T3752)

TGGCCTCGAGCCAGATTCGGACGAGGACCAGGGATCGTGCAAGCAAGGAAGCAGCCCTGGGGTGACACCCAGCACGTACTCCTGTGACAGAGCCGAGCCCAGCCCAGCCCCGGGACGCTTCGCAGAGGAGTCGCGGGAGGGTCCAGCTCGCTGTCGCTGAACCGAGCCTGTTCCTGCGCCCAGTCTGCGGGGGACCCTTGAACCGTAGAGACCCCAAGACCACCGAGCTGATGGCATCTTCCACTCCTTCGCCTGCAACCTCCTCGAACGCGGGAGCGGATCCTAATACTACCAACCTGCGCCCTACAACGTATGATACTTGGTGTGGCGTAGCCCATGGATGCACCAGAAAACTGGGCCTGAAGATCTGTGGCTTCTTACAAAGGACCAATAGCCTGGAAGAGAAGAGTCGTCTTGTGAGCGCCTTCAGGGAGAGGCAGTCCTCCAAGAACCTGCTTTCCTGTGAAAACAGTGACCAGGGTGCCCGCTTCCGGCGCACAGAGACCGACTTCTCCAACCTGTTTGCTCAAGATCTGCTTCCAGCTAAGA

*Vglut1* probe sense sequence (Allen Brain Atlas, NCBI Accession NM_182993.1):

CAGAGCCGGAGGAGATGAGCGAGGAGAAGTGTGGCTTTGTTGGCCACGACCAGCTGGCTGGCAGTGACGAAAGTGAAATGGAGGACGAGGCTGAGCCCCCAGGGGCGCCCCCCGCGCCGCCTCCGTCCTACGGGGCCACACACAGCACAGTGCAGCCTCCGAGGCCCCCGCCCCCTGTCCGGGACTACTGACCACGGGCCTCCCACTGTGGGGCAGTTTCCAGGACTTCCACTCCATACACCTCTAGCCTGAGCGGCAGTGTCGAGGAACCCCACTCCTCCCCTGCCTCAGGCTTAAGATGCAAGTCCTCCCTTGTTCCCAGTGCTGTCCGACCAGCCCTCTTTCCCTCTCAACTGCCTCCTGCGGGGGGTGAAGCTGCACACTAGCAGTTTCAAGGATACCCAGACTCCCCTGAAAGTCGTTCTCCGCTTGTTTCTGCCTGTGTGGGCTCAAATCTCCCCTTTGAGGGCTTTATTTGGAGGGACAGTTCAACCTCTTCCTCTCTTGTGGTTTTGAGGTTTCACCCCTTCCCCCAAGACCCCAGGGATTCTCAGGCTACCCCGAGATTATTCAGGTGGTCCCCTACTCAGAAGACTTCATGGTCGTCCTCTATTAGTTTCAAGGCTCGCCTAACCAATTCTACATTTTTCCAAGCTGGTTTAACCTAACCACCAATGCCGCCGTTCCCAGGACTGATTCTCACCAGCGTTTCTGAGGGA

*Vglut2* probe sequence (Allen Brain Atlas, NCBI Accession NM_080853.2):

CCAAATCTTACGGTGCTACCTCACAGGAGAATGGAGGCTGGCCTAACGGCTGGGAGAAAAAGGAAGAATTTGTGCAAGAAGGTGCGCAAGACGCGTACACCTATAAGGACCGAGATGATTATTCATAACGATGCTAGTTGCTGGATTCATTTGTAGTGTTTGTGAATCAATTAATTGTGATTGCACAAAAATAATTTTAAAAATGTGGTGTGAACATGTAAACATATCAACCAAGCAAGTCTTGCTGTTCAAAAACAAAAACAAAAAAATCTGAATTCAAAACAGACCATGAGATTCCCATCAAGTGCAATCTGTGGCAGTTGTCACGTTATGCCGTCTTCATTCAGGCCATTTGTCCTTTCGTTTGTGATTTAAAGGTTTCCTGTAGAAATAAGTAGGTATTCGTTGGACCCATCACCATTTTAGAGAGCACAACTACAACAGTTGGCACATGTCATCCTACAGAAGTTAGGAAGCCAAAGCTACTGGATCATGCAAACTGCACTTATTTATTACACTGGACTGCAAACTATCCCAGGGAAAGCCTGTCTAGAGACATAGTGGAACAGGAAAGATGGCT

*tdTomato* probe sequence (Allen Brain Atlas, NCBI Accession AY678269.1):

ATCAAAGAGTTCATGCGCTTCAAGGTGCGCATGGAGGGCTCCATGAACGGCCACGAGTTCGAGATCGAGGGCGAGGGCGAGGGCCGCCCCTACGAGGGCACCCAGACCGCCAAGCTGAAGGTGACCAAGGGCGGCCCCCTGCCCTTCGCCTGGGACATCCTGTCCCCCCAGTTCATGTACGGCTCCAAGGCGTACGTGAAGCACCCCGCCGACATCCCCGATTACAAGAAGCTGTCCTTCCCCGAGGGCTTCAAGTGGGAGCGCGTGATGAACTTCGAGGACGGCGGTCTGGTGACCGTGACCCAGGACTCCTCCCTGCAGGACGGCACGCTGATCTACAAGGTGAAGATGCGCGGCACCAACTTCCCCCCCGACGGCCCCGTAATGCAGAAGAAGACCATGGGCTGGGAGGCCTCCACCGAGCGCCTGTACCCCCGCGACGGCGTGCTGAAGGGCGAGATCCACCAGGCCCTGAAGCTGAAGGACGGCGGCCACTACCTGGTGGAGTTCAAGACCATCTACATGGCCAAGAAGCCCGTGCAACTGCCCGGCTACTACTACGTGGACACCAAGCTGGACATCACCTCCCACAACGAGGACTACACCATCGTGGAA
