# Supplementary figures and images for: GABAergic/Glycinergic and Glutamatergic Neurons Mediate Distinct Neurodevelopmental Phenotypes of STXBP1 Encephalopathy
Source: J Neurosci. 2024 Feb 15;44(14):e1806232024. doi: 10.1523/JNEUROSCI.1806-23.2024 (PMC10993039; doi:10.1523/JNEUROSCI.1806-23.2024)

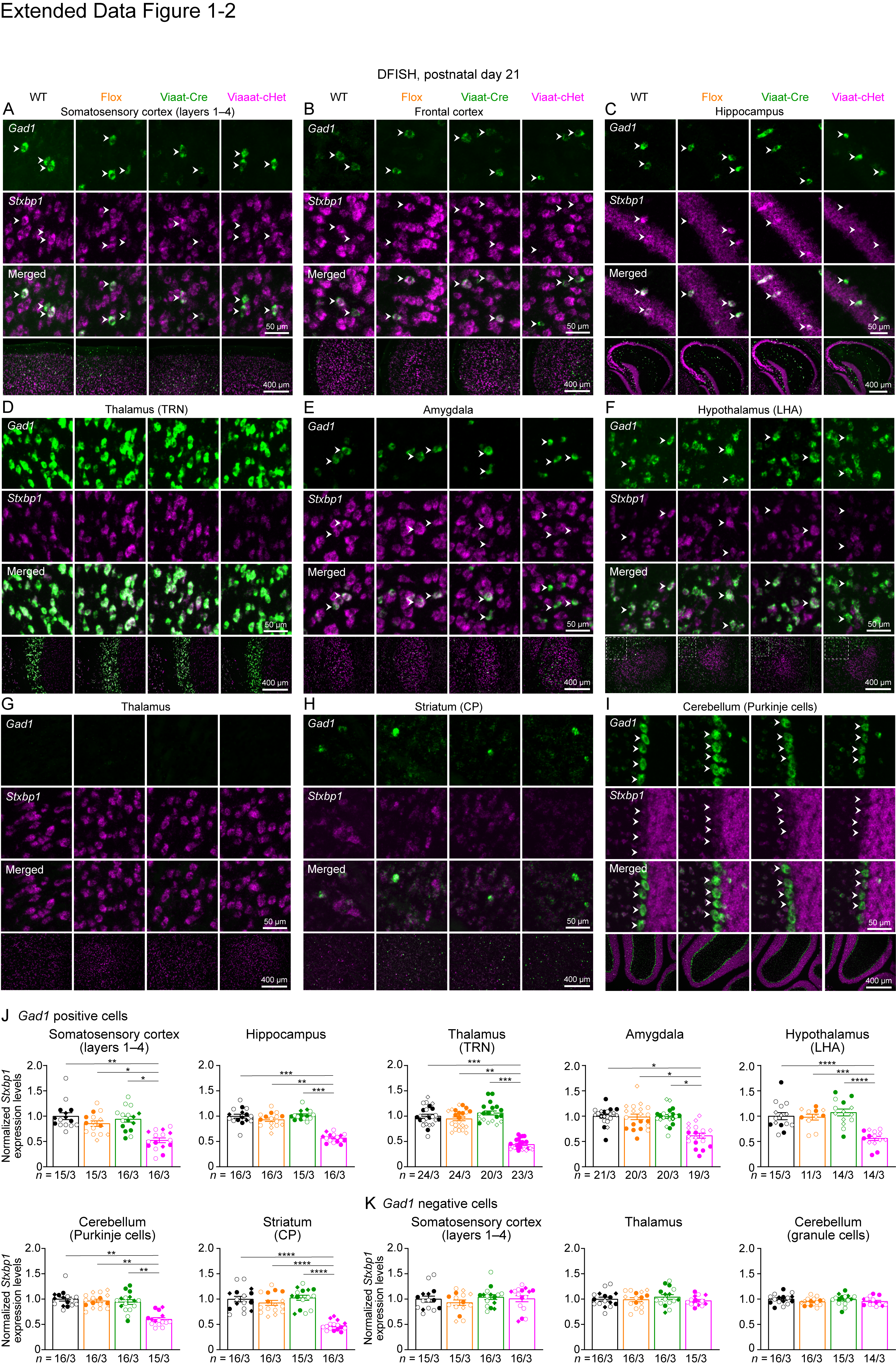

Supplement: Extended Data Figure 1-2 — Reduction of Stxbp1 mRNA levels specifically in GABAergic neurons of Viaat-cHet mice. (A) Representative fluorescence images from brain sections labeled by ISH probes against Stxbp1 and Gad1. The bottom row shows the layers 1–4 of the somatosensory cortex, and the top three rows show the individual cells from this region. Arrows heads indicate Gad1-positive cells. (B–I) Similar to (A), but for other brain regions indicated on the top of each panel. TRN, thalamic reticular nucleus; LHA, lateral hypothalamic area; CP, caudoputamen. (J) Summary data of normalized Stxbp1 mRNA levels in Gad1­-positive cells from different brain regions. Stxbp1 levels were normalized by the average Stxbp1 levels of WT brain sections that were stained and imaged in parallel. The Stxbp1 levels of Viaat-cHet mice were reduced in all brain regions. Different shapes of symbols represent different mice (3 mice per genotype, filled circles for 1 male and open circles and diamonds for 2 females), and each symbol represents one brain section. (K) Similar to (J), but for Gad1­-negative cells from different brain regions. The Stxbp1 levels of Gad1­-negative cells in Viaat-cHet mice were normal. Data are mean ± s.e.m. * P < 0.05, ** P < 0.01, *** P < 0.001, **** P < 0.0001. Download Extended Data Figure 1-2, TIF file. [file jneuro-44-e1806232024-s002.tif]

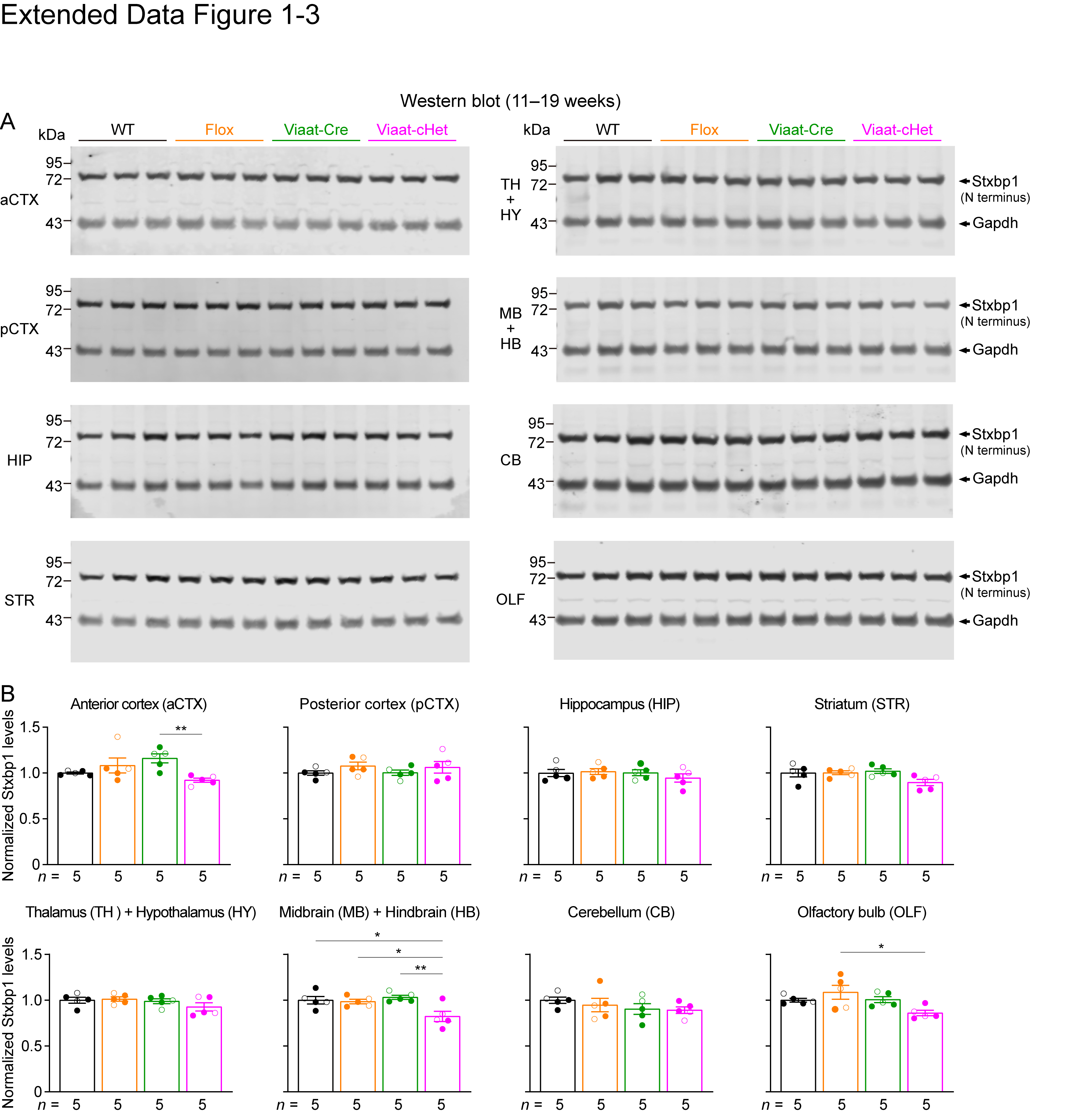

Supplement: Extended Data Figure 1-3 — Mild reduction of Stxbp1 protein levels in Viaat-cHet mice. (A) Representative Western blots of proteins from different brain regions of WT, Flox, Viaat-Cre, and Viaat-cHet mice at the age of 11–19 weeks. Stxbp1 was detected by an antibody recognizing its N terminus. Gapdh, a housekeeping protein as loading control. (B) Summary data of normalized Stxbp1 protein levels. Stxbp1 levels were first normalized by the Gapdh levels and then by the average Stxbp1 levels of all WT mice from the same blot. Each filled (male) or open (female) circle represents one mouse. Data are mean ± s.e.m. * P < 0.05, ** P < 0.01. Download Extended Data Figure 1-3, TIF file. [file jneuro-44-e1806232024-s003.tif]

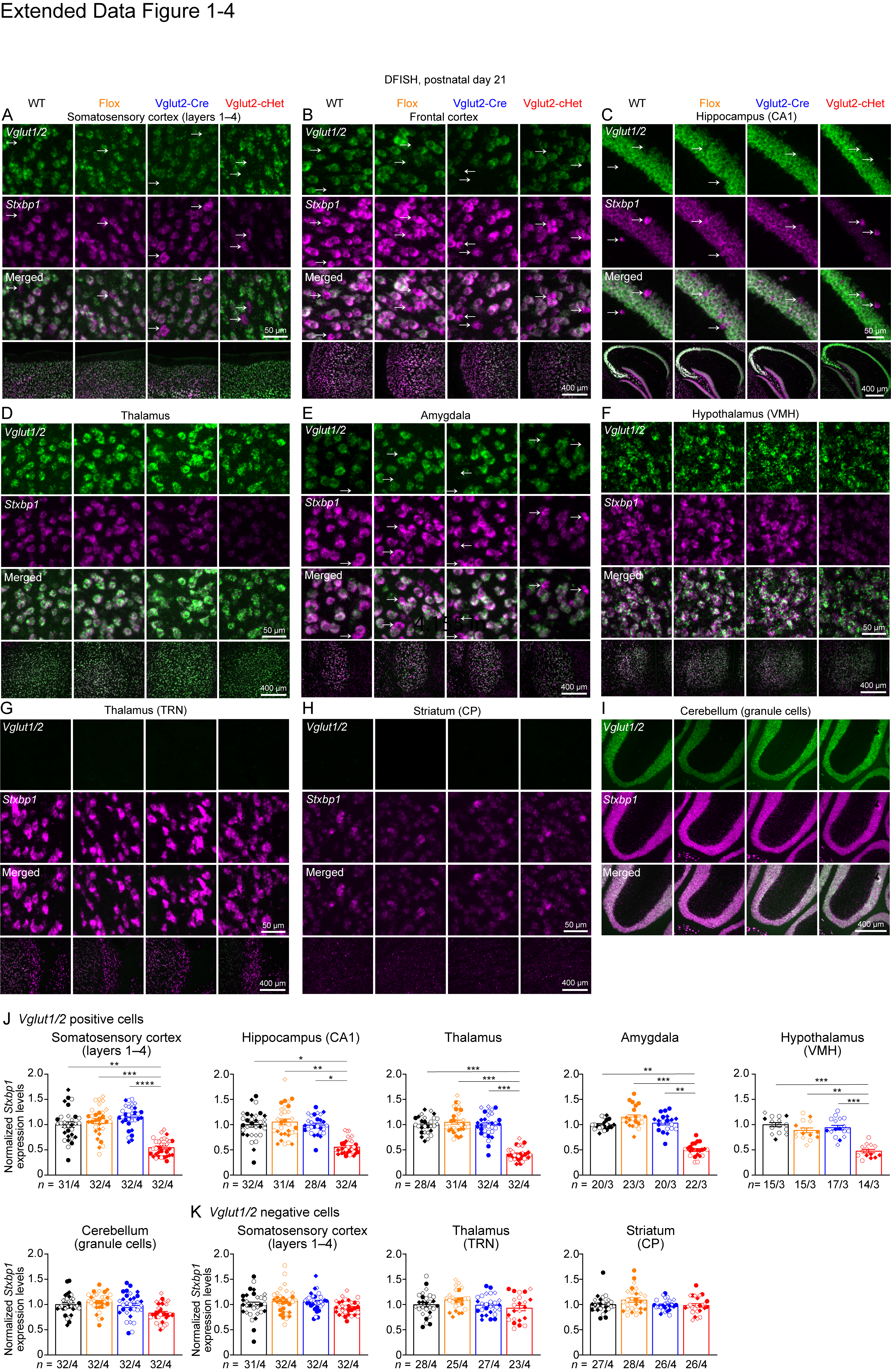

Supplement: Extended Data Figure 1-4 — Reduction of Stxbp1 mRNA levels specifically in glutamatergic neurons of Vglut2-cHet mice. (A) Representative fluorescence images from brain sections labeled by ISH probes against Stxbp1 and Vglut1/2. The bottom row shows the layers 1–4 of the somatosensory cortex, and the top three rows show the individual cells from this region. Arrows indicate Vglut1/2-negative cells. (B–I) Similar to (A), but for other brain regions indicated on the top of each panel. VMH, ventromedial hypothalamic nucleus; TRN, thalamic reticular nucleus; CP, caudoputamen. (J) Summary data of normalized Stxbp1 mRNA levels in Vglut1/2­-positive cells from different brain regions. Stxbp1 levels were normalized by the average Stxbp1 levels of WT brain sections that were stained and imaged in parallel. The Stxbp1 levels of Vglut2-cHet mice were reduced in most brain regions except cerebellar granule cells. Different shapes of symbols represent different mice (4 mice per genotype, filled circles and diamonds for 2 males and open circles and diamonds for 2 females), and each symbol represents one brain section. (K) Similar to (J), but for Vglut1/2­-negative cells from different brain regions. The Stxbp1 levels of Vglut1/2­-negative cells in Vglut2-cHet mice were normal. Data are mean ± s.e.m. * P < 0.05, ** P < 0.01, *** P < 0.001, **** P < 0.0001. Download Extended Data Figure 1-4, TIF file. [file jneuro-44-e1806232024-s004.tif]

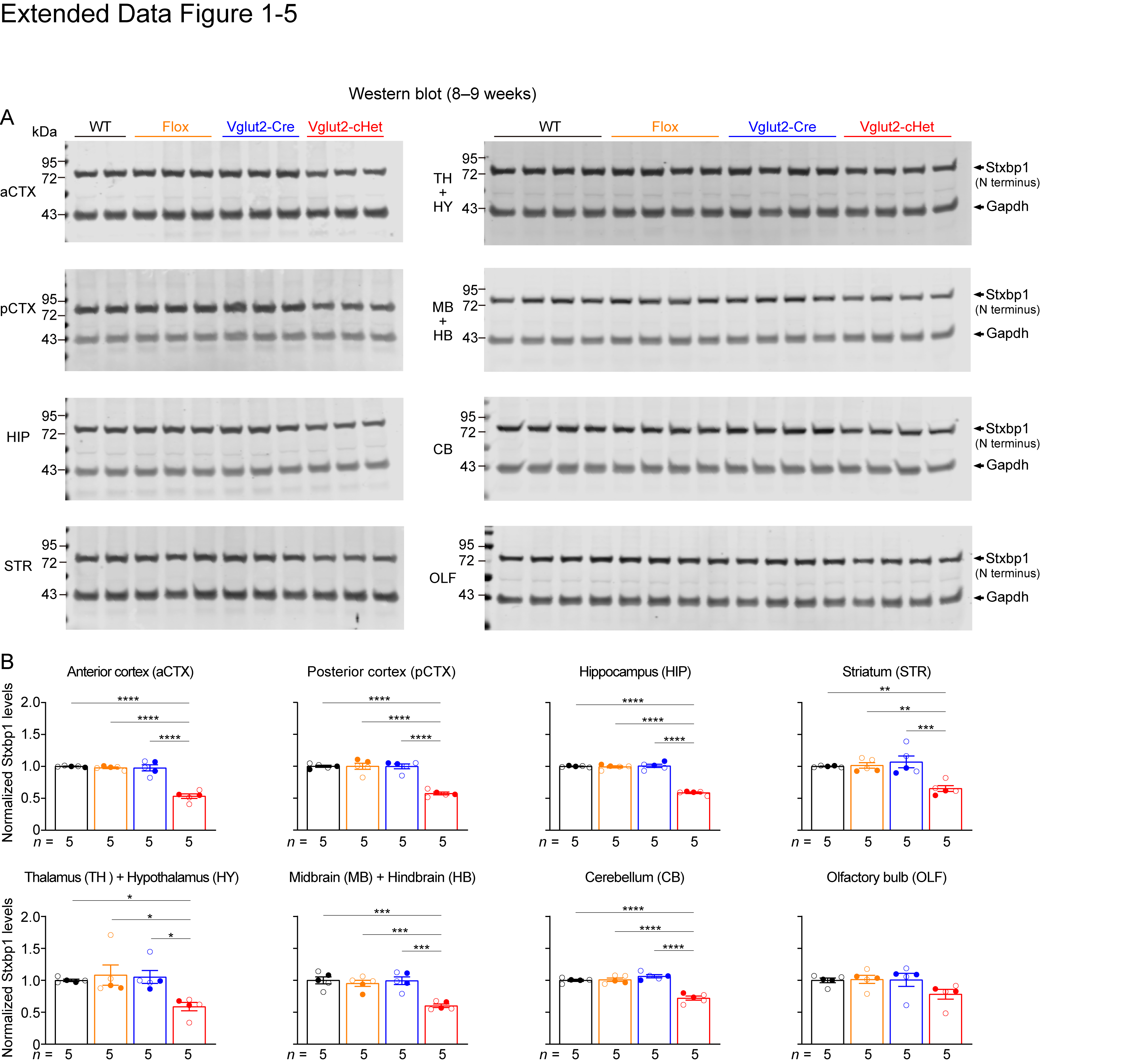

Supplement: Extended Data Figure 1-5 — Reduction of Stxbp1 protein levels in Vglut2-cHet mice. (A) Representative Western blots of proteins from different brain regions of WT, Flox, Vglut2-Cre, and Vglut2-cHet mice at the age of 8–9 weeks. Stxbp1 was detected by an antibody recognizing its N terminus. Gapdh, a housekeeping protein as loading control. (B) Summary data of normalized Stxbp1 protein levels. Stxbp1 levels were first normalized by the Gapdh levels and then by the average Stxbp1 levels of all WT mice from the same blot. Each filled (male) or open (female) circle represents one mouse. Data are mean ± s.e.m. * P < 0.05, ** P < 0.01, *** P < 0.001, **** P < 0.0001. Download Extended Data Figure 1-5, TIF file. [file jneuro-44-e1806232024-s005.tif]

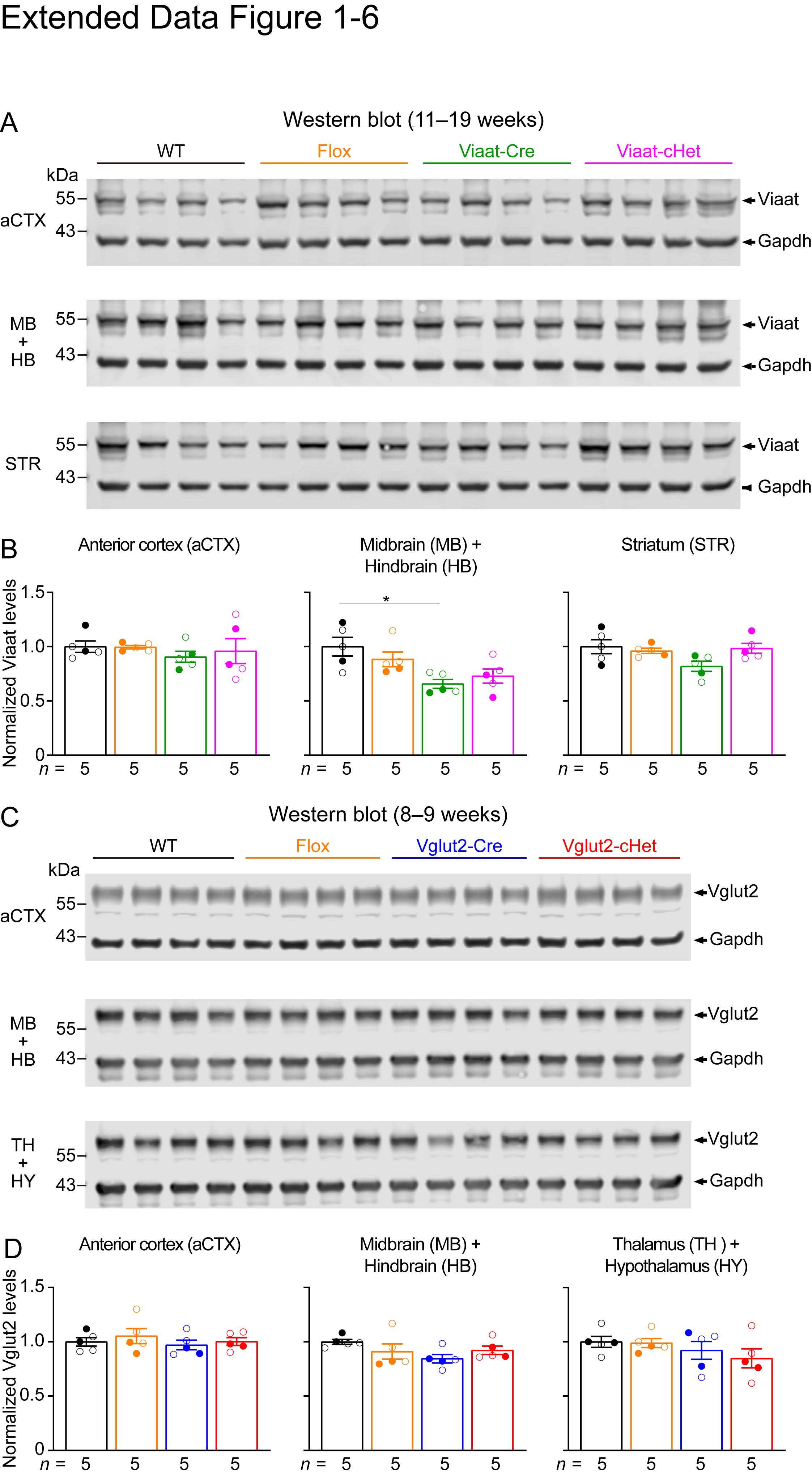

Supplement: Extended Data Figure 1-6 — Normal Viaat and Vglut2 protein levels in Viaat-cHet and Vglut2-cHet mice, respectively. (A) Representative Western blots of Viaat proteins from different brain regions of WT, Flox, Viaat-Cre, and Viaat-cHet mice at the age of 11–19 weeks. Gapdh, a housekeeping protein as loading control. (B) Summary data of normalized Viaat protein levels. Viaat levels were first normalized by the Gapdh levels and then by the average Viaat levels of all WT mice from the same blot. (C,D) Similar to (A,B), but for Vglut2 protein levels in WT, Flox, Vglut2-Cre, and Vglut2-cHet mice at the age of 8–9 weeks. Each filled (male) or open (female) circle represents one mouse. Data are mean ± s.e.m. * P < 0.05. Download Extended Data Figure 1-6, TIF file. [file jneuro-44-e1806232024-s006.tif]

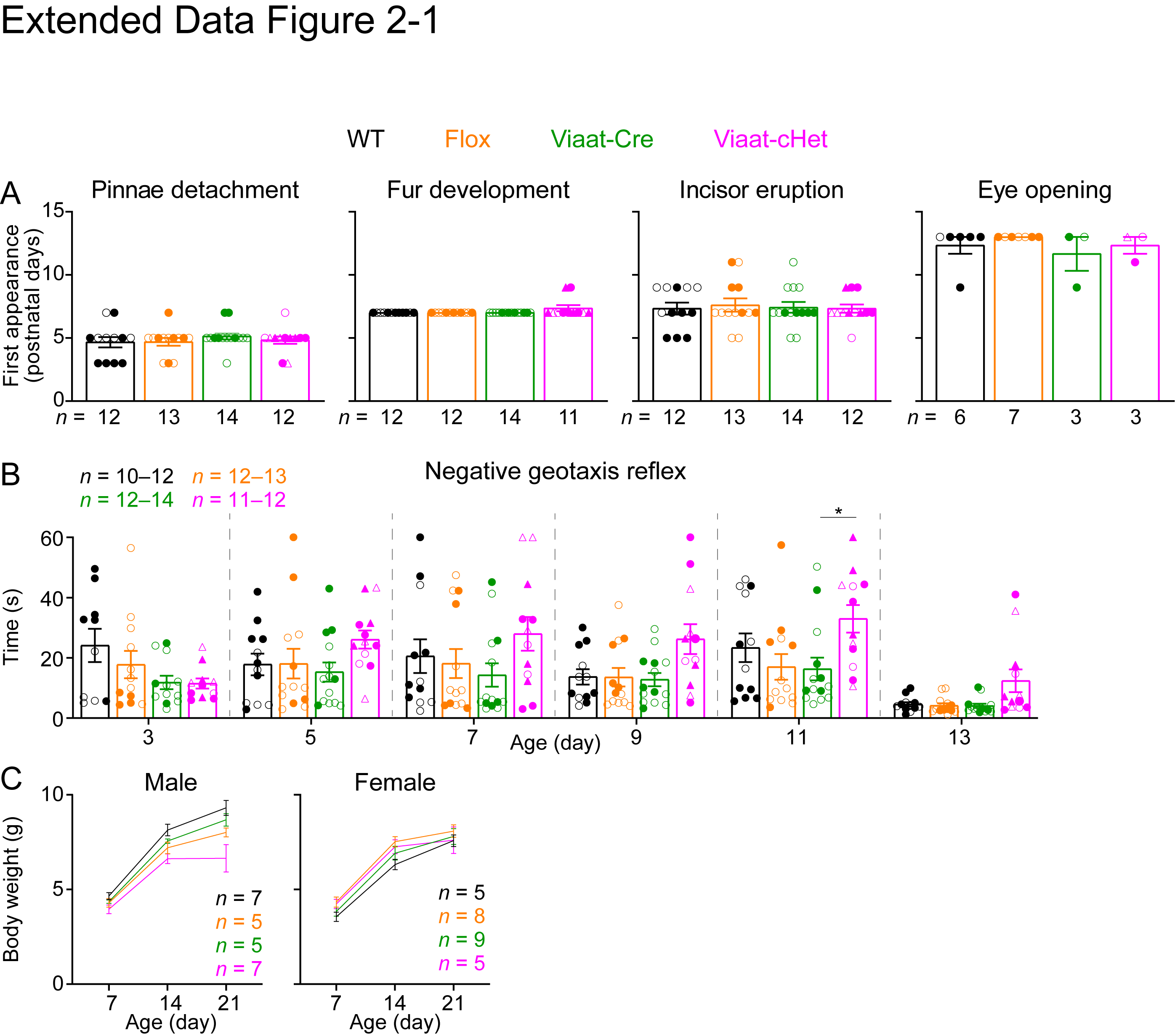

Supplement: Extended Data Figure 2-1 — Developmental milestones of Viaat-cHet mice. (A) The postnatal days when the developmental milestones including pinnae detachment, fur development, incisor eruption, and eye opening were first observed. Note, due to illness of the experimenter, the eye opening days of a subset of mice were not recorded. (B) When placing on an inclined plane with the head facing downwards, the amount of time it took for the pup to orientate itself with its head facing upwards as a function of age. The negative geotaxis reflex was normal in Viaat-cHet mice. Note, the filled (male) and open (female) triangles represent those pups that later died between P14–21. (C) Body weight as a function of age. The body weight of Viaat-cHet mice was not significantly different from that of control mice during the first 3 postnatal weeks. For different panels, the numbers and ages of tested mice are indicated in the figures. Each filled (male) or open (female) circle represents one mouse. Data are mean ± s.e.m. * P < 0.05. Download Extended Data Figure 2-1, TIF file. [file jneuro-44-e1806232024-s008.tif]

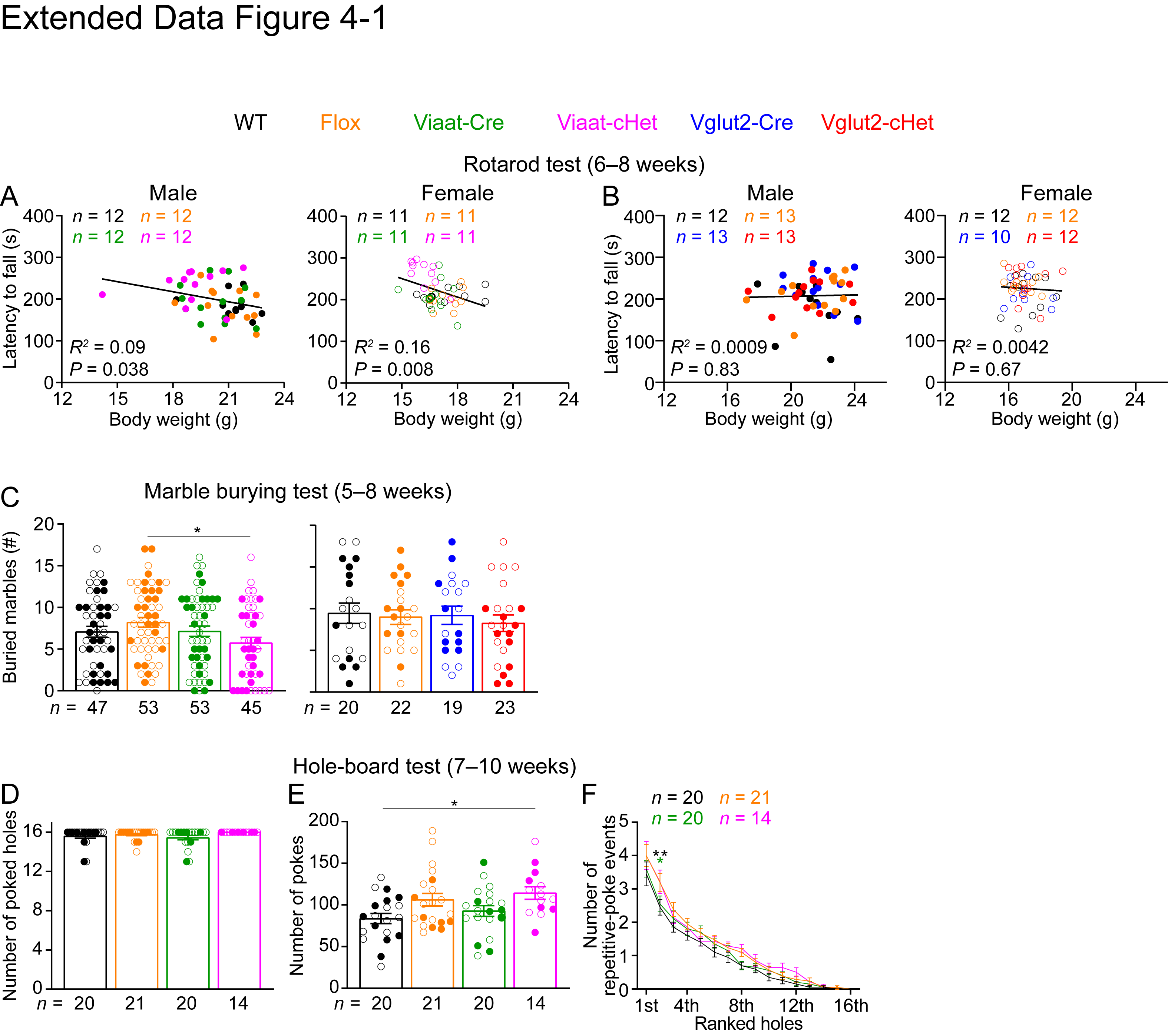

Supplement: Extended Data Figure 4-1 — Viaat-cHet mice do not show heightened repetitive and stereotyped behaviors. (A) The average latency to fall from the rotating rod across all trials as a function of body weight for Viaat-cHet and their control mice. There are weak but statistically significant negative correlations between the latency and body weight for both male and female mice. (B) Similar to (A), but for Vglut2-cHet and their control mice. There are no significant correlations. (C) The numbers of buried marbles (i.e., at least 50% of the marble is covered) for Viaat-cHet, Vglut2-cHet, and their control mice. (D–F) In the hole-board test, the total numbers of holes that were poked (D) and the total numbers of nose pokes (E) by Viaat-cHet were similar to those by the control mice. The holes were also ranked according to the numbers of repetitive-poke events (i.e., with 2 or more consecutive pokes) in each hole (F). The statistical significance between Viaat-cHet and WT or Viaat-Cre mice is indicated by black or green asterisks, respectively. For different panels, the numbers and ages of tested mice are indicated in the figure. Each filled (male) or open (female) circle represents one mouse. Data are mean ± s.e.m. * P < 0.05. Download Extended Data Figure 4-1, TIF file. [file jneuro-44-e1806232024-s012.tif]

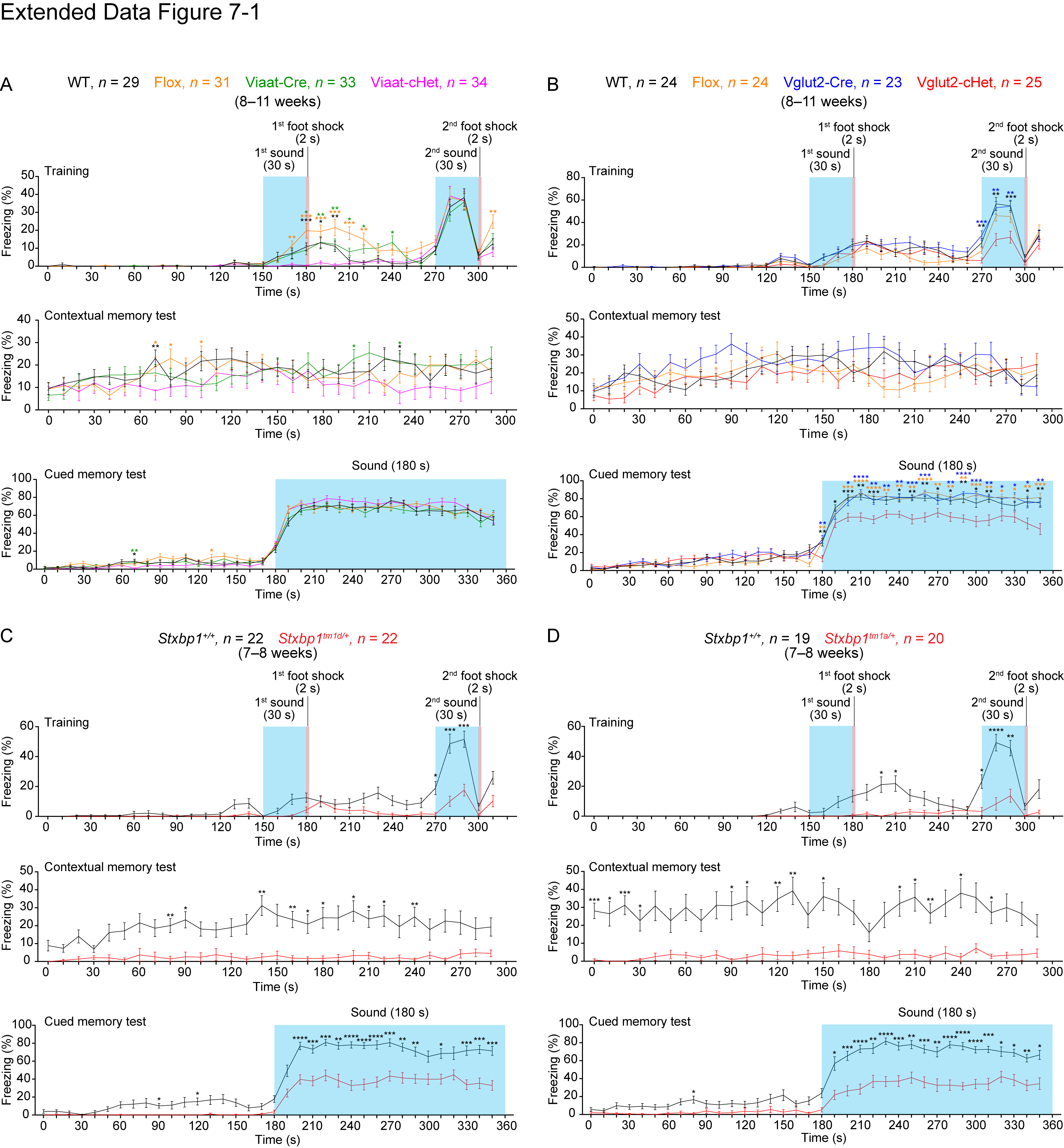

Supplement: Extended Data Figure 7-1 — The time courses of freezing behaviors in fear conditioning. (A) The freezing behaviors of Viaat-cHet and control mice as a function of time during training (upper panel), contextual memory test (middle panel), and cued memory test (lower panel) conducted 24 hours after training. During training, Viaat-cHet mice did not freeze as much as the control mice after the first and second sound presentations but froze similarly in response to the second sound presentation. The statistical significance between Viaat-cHet and WT, Flox, or Viaat-Cre mice is indicated by black, orange, or green asterisks, respectively. (B) Similar to (A), but for Vglut2-cHet and control mice. During training, Vglut2-cHet mice did not freeze as much as the control mice in response to the second sound presentation. The statistical significance between Vglut2-cHet and WT, Flox, or Vglut2-Cre mice is indicated by black, orange, or blue asterisks, respectively. (C,D) Similar to (A), but for Stxbp1tm1d/+, Stxbp1tm1a/+, and control mice. During training, Stxbp1tm1d/+ and Stxbp1tm1a/+ mice did not freeze as much as the control mice after the first and second sound presentations and in response to the second sound presentation. For different panels, the numbers and ages of tested mice are indicated in the figure. Data are mean ± s.e.m. * P < 0.05, ** P < 0.01, *** P < 0. 001, **** P < 0. 0001. Download Extended Data Figure 7-1, TIF file. [file jneuro-44-e1806232024-s013.tif]

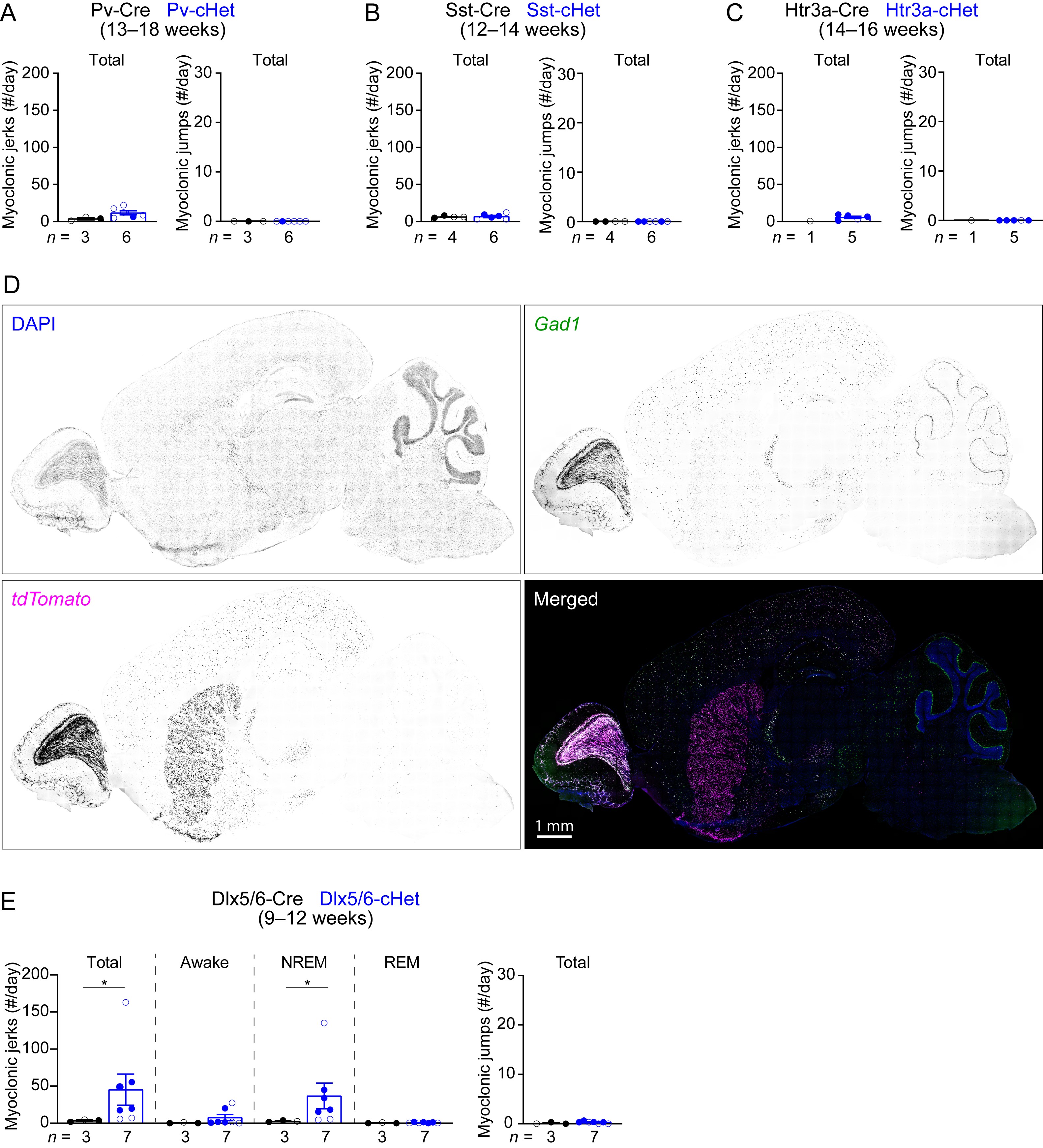

Supplement: Extended Data Figure 8-4 — Stxbp1 haploinsufficiency in subtypes of GABAergic neurons. (A) Summary data showing the total frequencies of myoclonic jerks (left panel) and jumps (right panel) from Pv-cHet (Stxbp1f/+;PvCre/+) and control Pv-Cre (PvCre/+) mice. (B,C) Similar to (A), but for Sst-cHet (Stxbp1f/+;SstCre/+) and control Sst-Cre (SstCre/+) mice (B) and Htr3a-cHet (Stxbp1f/+;Htr3a-CreTg/+) and control Htr3a-Cre (Htr3a-Cre Tg/+) mice (C). (D) Representative fluorescence images of a sagittal brain section from Dlx5/6-CreTg/+;Rosa26tdTomato/+ mice (n = 2, P92 and P101) labeled by ISH probes against tdTomato and Gad1 and a nuclear marker DAPI. tdTomato expression is largely restricted to forebrain Gad1-positive cells. (E) Summary data showing the total frequencies of myoclonic jerks and the frequencies in different behavioral states (left panel) and total frequencies of myoclonic jumps (right panel) from Dlx5/6-cHet (Stxbp1f/+;Dlx5/6-CreTg/+) and control Dlx5/6-Cre (Dlx5/6-CreTg/+) mice. For different panels, the numbers and ages of recorded mice are indicated in the figure. Each filled (male) or open (female) circle represents one mouse. Data are mean ± s.e.m. * P < 0.05. Download Extended Data Figure 8-4, TIF file. [file jneuro-44-e1806232024-s020.jpg]
