# Supplementary material for: GABAergic/Glycinergic and Glutamatergic Neurons Mediate Distinct Neurodevelopmental Phenotypes of STXBP1 Encephalopathy
Source: J Neurosci. 2024 Feb 15;44(14):e1806232024. doi: 10.1523/JNEUROSCI.1806-23.2024 (PMC10993039; doi:10.1523/JNEUROSCI.1806-23.2024)
Supplement: Extended data Table 9-1 — Phenotypic comparison of human patients and different mouse models. The phenotyping tests in different mouse models (the second column) are grouped based on the clinical features of STXBP1 encephalopathy (the first column). The results of the phenotyping tests from different mouse models are compared in the table. Download Extended data Table 9-1, DOCX file. [file jneuro-44-e1806232024-s021.docx]

**Phenotypic comparison of *Stxbp1* constitutive and cell-type specific haploinsufficiency mouse models**

| Human patient phenotypes  (% of patients^1^) | Mouse phenotyping tests | *Stxbp1* mouse models and phenotypes^2^ | | | | | |
| --- | --- | --- | --- | --- | --- | --- | --- |
|  |  | *Stxbp1^tm1d/+^* | *Stxbp1^f/+^;*  *Emx1^Cre/+^* | *Stxbp1^f/+^;*  *Viaat-Cre^Tg/+^* | *Stxbp1^f/+^;*  *Gad2^Cre/+^* | *Stxbp1^f/+^;*  *Viaat^Cre/+^* | *Stxbp1^f/+^;*  *Vglut2^Cre/+^* |
|  |  | Chen et al., 2020 | Miyamoto et al., 2017; 2019 | Miyamoto et al., 2017; 2019 | Kovačević et al., 2018 | This paper | This paper |
| – | Stxbp1 protein or mRNA reduction in targeted brain tissues | Protein (Western blot):  40–50% in cortex, hippocampus, thalamus+ hypothalamus, striatum, midbrain+ hindbrain;  30% in cerebellum;  20% in olfactory bulb | Protein (Western blot):  40% in cortex, hippocampus | Protein (Western blot):  little change in cortex, hippocampus | – | mRNA (DFISH)^3^:  51–56% reduction in thalamic reticular nucleus and striatum;  37–45% in frontal cortex, somatosensory cortex, hippocampus, hypothalamus (LHA), amygdala, and cerebellar Purkinje cells  Protein (Western blot)^3^: 11–18% reduction in anterior cortex, striatum, olfactory bulb, and midbrain+ hindbrain; 0–10% in posterior cortex, hippocampus, thalamus+ hypothalamus, and cerebellum | mRNA (DFISH)^3^:  50–55% reduction in frontal cortex, somatosensory cortex, thalamus, amygdala;  46–48% in hippocampus and hypothalamus (VMH);  18% in cerebellar granular cells  Protein (Western blot)^3^: 30–45% reduction in anterior cortex, posterior cortex, hippocampus, striatum, thalamus+ hypothalamus, and midbrain+ hindbrain; 21-28% in olfactory bulb and cerebellum |
| – | Early lethality | Yes | No | No | Yes | Yes | No |
| Developmental delay (86%) | Body weight | Yes | No | No | – | Yes | No |
|  | Surface righting reflex | – | – | – | – | Yes | – |
|  | Negative geotaxis reflex | – | – | – | – | No | – |
| Epilepsy (89%) | Video-EEG/EMG | SWDs, myoclonic seizures | SWDs | Twitches and jumps | Epileptiform activity | Myoclonic seizures | SWDs |
| Intellectual disability (>95%) | Novel object recognition | Yes | – | – | – | No | No |
|  | Contextual fear | Yes | Yes | No | – | Yes | No |
|  | Cued fear | Yes | – | – | – | No | Yes |
| Motor deficits  (92%) | Hindlimb clasping | Yes | – | – | – | Yes | Yes |
|  | Foot slip | Yes | – | – | – | Yes | No |
|  | Vertical pole | Yes | – | – | – | Yes | No |
|  | Rotarod | No^4^ | – | – | – | No^4^ | No |
| Hyperactivity (4%) | Open-field | Yes | No | No | – | Yes | No |
| Autistic traits (20%) | Three-chamber | No | – | – | – | No | No |
|  | Hole-board | Yes | – | – | – | No | – |
| Aggressive behavior (3.4%) | Resident-intruder | Yes | No | No | – | Yes | No |
| Anxiety  (27%) | Elevated plus maze | Yes | – | – | – | Yes | No |
|  | Open-field | Yes | No | No | – | Yes | Yes |
| – | Nest building | Yes | – | – | – | Yes | No |
|  | Marble burying | Yes | – | – | – | No | No |
|  | Startle reactivity | No | – | – | – | No | No |
|  | Pre-pulse inhibition | No | – | – | – | No | No |
|  | Hot plate | No | – | – | – | No | No |

^1^Percentage is based on Stamberger et al., 2016, Suri et al., 2017, and Xian et al., 2021.

^2^For the phenotypes of mouse models, “Yes” indicates a statistical difference between mutant and control mice and “No” indicates no statistical differences detected between mutant and control mice.

^3^The changes of mRNA or protein levels in Viaat-cHet and Vglut2-cHet mice were calculated relative to the average levels of those in the corresponding Flox and Cre control mice.

^4^*Stxbp1^tm1d/+^* and *Stxbp1^f/+^;Viaat^Cre/+^* mice performed better than control mice at the age of 6–8 weeks.
